# LINE-1 promotes tumorigenicity and exacerbates tumor progression via stimulating metabolism reprogramming in non-small cell lung cancer

**DOI:** 10.1186/s12943-022-01618-5

**Published:** 2022-07-16

**Authors:** Zeguo Sun, Rui Zhang, Xiao Zhang, Yifei Sun, Pengpeng Liu, Nancy Francoeur, Lei Han, Wan Yee Lam, Zhengzi Yi, Robert Sebra, Martin Walsh, Jinpu Yu, Weijia Zhang

**Affiliations:** 1 Department of Medicine, Icahn School of Medicine at Mount Sinai, New York City, NY USA; 2 Cancer Molecular Diagnostics Core, Tianjin Medical University Cancer Institute & Hospital, National Clinical Research Center of Cancer, Key Laboratory of Cancer Prevention and Therapy, Key Laboratory of Cancer Immunology and Biotherapy, Tianjin’s Clinical Research Center for Cancer, Tianjin, China; 3 Department of Pharmacological Sciences, Icahn School of Medicine at Mount Sinai, New York City, NY USA; 4 Department of Genetics and Genomic Sciences, Icahn School of Medicine at Mount Sinai, New York City, NY USA

**Keywords:** LINE-1, Transposable element, NSCLC, LUSC, LUAD, FGGY, Metabolic

## Abstract

**Background:**

Long Interspersed Nuclear Element-1 (LINE-1, L1) is increasingly regarded as a genetic risk for lung cancer. Transcriptionally active LINE-1 forms a L1-gene chimeric transcript (LCTs), through somatic L1 retrotransposition (LRT) or L1 antisense promoter (L1-ASP) activation, to play an oncogenic role in cancer progression.

**Methods:**

Here, we developed Retrotransposon-gene fusion estimation program (ReFuse), to identify and quantify LCTs in RNA sequencing data from TCGA lung cancer cohort (*n* = 1146) and a single cell RNA sequencing dataset then further validated those LCTs in an independent cohort (*n* = 134). We next examined the functional roles of a cancer specific LCT (*L1-FGGY*) in cell proliferation and tumor progression in LUSC cell lines and mice.

**Results:**

The LCT events correspond with specific metabolic processes and mitochondrial functions and was associated with genomic instability, hypomethylation, tumor stage and tumor immune microenvironment (TIME). Functional analysis of a tumor specific and frequent LCT involving *FGGY* (*L1-FGGY*) reveal that the arachidonic acid (AA) metabolic pathway was activated by the loss of *FGGY* through the *L1-FGGY* chimeric transcript to promote tumor growth, which was effectively targeted by a combined use of an anti-HIV drug (NVR) and a metabolic inhibitor (ML355). Lastly, we identified a set of transcriptomic signatures to stratify the LUSC patients with a higher risk for poor outcomes who may benefit from treatments using NVR alone or combined with an anti-metabolism drug.

**Conclusions:**

This study is the first to characterize the role of L1 in metabolic reprogramming of lung cancer and provide rationale for L1-specifc prognosis and potential for a therapeutic strategy for treating lung cancer.

**Trial registration:**

Study on the mechanisms of the mobile element *L1-FGGY* promoting the proliferation, invasion and immune escape of lung squamous cell carcinoma through the 12-LOX/Wnt pathway, Ek2020111. Registered 27 March 2020 ‐ Retrospectively registered.

**Supplementary Information:**

The online version contains supplementary material available at 10.1186/s12943-022-01618-5.

## Introduction

The Long Interspersed Nuclear Element-1 (LINE-1, L1), representing a family of non-long-terminal repeat (LTR) transposable elements (TEs), constitute 17% of the human genome with ~ 500,000 copies widely distributed in the genome [1]. Transcriptionally active L1s can propagate themselves and insert into a gene locus in the genome via the reverse transcription of the transposon [2] (called L1 retrotransposition (LRT)). In other cases, through the activation of the L1 antisense promoter (L1-ASP), the L1s within an intronic region can also be transcribed into the adjacent exon of a gene through a splicing site to generate L1-gene chimeric transcripts (LCTs) which might affect the expression or functions of a gene [3, 4]. The LCTs through L1-ASP activation were reported to have abnormally high expression in most cancer tissues and be associated with oncogenic activity [5–7].

Increased L1 activity is associated with cancer, neural degenerative diseases and many other diseases. Therefore, the detection of LRTs and trans-splicing events is key to understand how L1s can alter gene expression or function leading to disease development and progression. Many strategies have been performed to observe LRTs at the DNA level based on the whole exome or genome sequencing and L1-targeted sequencing [8–12]. While DNA sequencing may lack the resolution to capture trans-splicing events and quantify the level of L1 activities at the transcriptional level, alternative approaches have begun to place some emphasis on developing informatic tools to detect LCTs based on short-read RNA sequencing data [13–15]. Because of technical limitations, such as requiring high sequencing depth for sequencing assembly or other restrictions, LCT events detected by these tools remain limited. In addition, these analytical tools rely on paired end sequencing data, making it difficult to infer from single cell sequencing data to study LCT events at a single cell level. Therefore, an analytical framework that allows for accurate detection of genome-wide LCTs at whole transcriptome or single cell level is needed in cancer biology to better interrogate the role LCTs play in oncogenesis.

Lung cancer remains among the most common cancer types which has estimated 1.6 million death each year [16] and the 5-year survival rate of only about 18.1%, mainly due to the late diagnosis of advanced disease [16]. Non-small cell lung cancer (NSCLC), with common subtypes as lung adenocarcinoma (LUAD) and lung squamous cell carcinoma (LUSC), account for ~ 85% of lung cancer patients [17]. Results from whole exome sequencing (WES) of tumor samples have shown that the NSCLC corresponds with higher L1 activity among different cancer types [18]. In our previous study, we detected 13 frequent and recurrent LCTs from RNA sequencing data of TCGA LUSC cohort by the DeFuse program, a gene fusion detection-based program with a limitation in genome-wide LCT detection [19]. We detect one of the most prominent tumor-specific LCTs, *L1-FGGY*, which is formed by transcription of L1 in the intron region into the exon 13 of FGGY through L1-ASP activation. Interestingly, *FGGY* is involved with arachidonic acid metabolism and regarded as a putative tumor suppressor gene. *L1-FGGY* interferes with the tumor suppressor function of *FGGY*, thereby, promotes cancer cell proliferation, invasion and accelerated tumor growth corresponding with a tumor microenvironment deplete of immune cell populations to forge a cytotoxic response to tumor cells. This implicates the role of L1 in altering tumor cell metabolism thus allowing for the evasion of immunity in NSCLC [19], thereby requiring further genome-wide LCT assessment and functional studies in tumorigenesis.

In this study, we generated a novel bioinformatic approach called, ReFuse (Retrotransposon-gene fusion estimation program), as a means to accurately detect LCTs at a genome-wide scale from both bulk and single cell RNA sequencing data of NSCLC with greater sensitivity. Our approach reveals that LCT frequently affect genes corresponding with mitochondrial biogenesis and energetics linked with overall metabolic capacities with the underlying oncogenic roles of *L1-FGGY* in driving metabolic reprogramming in NSCLC. Finally, we confirm that reverse transcriptase inhibitors can disrupt L1 activity that results in the metabolic programming as putative rationales for considering the treatment of patients observed with higher levels of L1 activity. Our study is the first to report a functional role of L1 activity resulting in the reprogramming of metabolism leading to changes in the tumor microenvironment leading to accelerated lung cancer progression. The intersection between LCTs and their corresponding targets in the human genome leading to increased oncogenesis could represent a promising prognosis biomarker and therapeutic target in NSCLC.

## Result

### LCTs are frequently detected in NSCLC and mainly involved in mitochondrial function and metabolic process

**Fig. 1:**
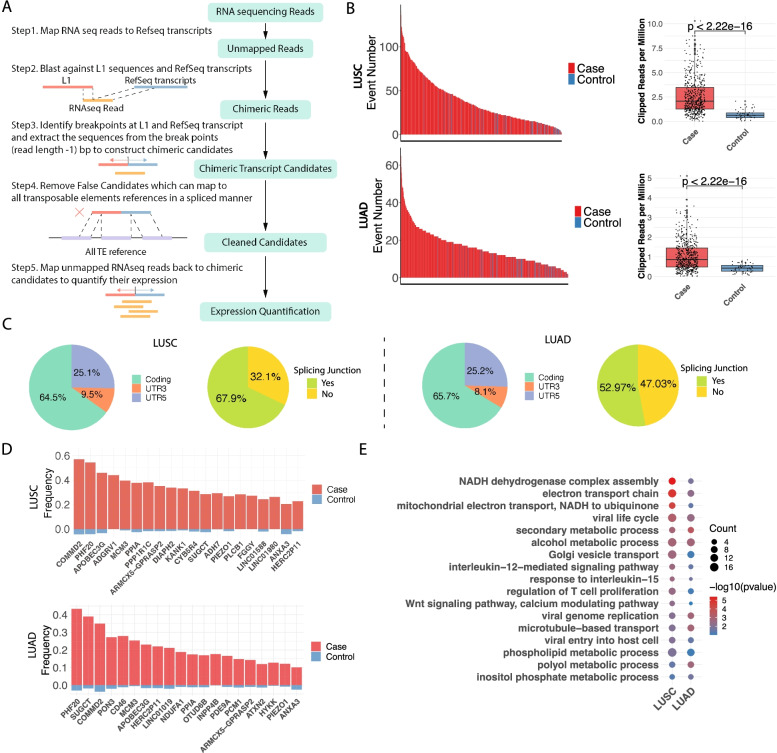
LCT Activity Identified by Refuse in LUSC and LUAD. **A** Workflow of the Refuse analysis. **B** Bar plot shows total number of LCT events detected and a box plot showing expression of LCT (normalized as reads per million) for each patient. Red represents tumor samples and blue represents normal controls. **C** The proportion of LCT events in coding, UTR3 or UTR5 region, and proportion of LCT events in splicing or non-splicing region in TCGA LUSC and LUAD separately. **D** The absolute frequency of tumor and normal samples containing LCT affected genes. Each gene was calculated separately and top 20 most frequently represented genes were showed. **E** Enriched GO terms for genes recurrently affected by LCT (in more than 2 samples), dot size represents the number of genes enriched in each term and the color scale represents the -log_10_(*p* value) of enrichment

To detect LCTs with a high level of accuracy at the genome-wide scale using Illumina-based short read data of RNA sequencing data derived from TCGA, we developed a local alignment-based program, ReFuse, to identify and quantify LCTs (Fig. 1A and Methods). Briefly, after filtering out the raw sequence reads that were fully aligned to RefSeq transcripts, we identified the chimeric sequence reads with one end partially aligned to RefSeq transcripts and the other end to L1 reference sequences. An LCT candidate was initially generated from a chimeric sequence read by combining sequences of RefSeq and L1 extended from the junction site with the length of read length minus one bp. All the LCTs were then clustered to remove duplicates to obtain the unique LCTs. The candidate LCTs were then aligned to all TE sequences from RepeatMasker database in a gap allowed manner to remove false positives, which could be a fusion of L1 and other TE sequences. All raw reads were aligned back to these unique LCTs to derive the final LCT candidates with a full alignment coverage by at least 5 reads and the expression value of LCT was quantified by the count of reads covering the LCT candidates (Fig. 1A and Methods).

We then applied ReFuse to bulk RNAseq data of LUSC (*n* = 551, 48 normal tissues) and LUAD (*n* = 595, 57 normal tissues) in TCGA cohort and an independent LUSC cohort from Tianjin Medical University Cancer Institute and Hospital (TJMUCH) as a validation (*n* = 134, 22 normal tissues) to identify genome-wide LCTs in all cohorts. We detected 1131 LCT events in 691 genes for LUSC and 725 LCT events in 545 genes for LUAD, respectively (Fig. 1B and Table S1). In the independent TJMUCH LUSC cohort, we observed a significant portion (351/848, 41.4%) of LCTs were overlapped with TCGA LUSC cohort (Figure S1A). There we confirmed the most frequent LCT event involving the intronic L1 transcription into exon13 in *FGGY* gene through ASP activation in both LUSC and LUAD, which was discovered in our previous study (Figure S2A). Another example demonstrated a novel frequent LCT event within non-coding gene *LINC01980*, which was formed by trans-splicing of intronic L1 into the 5th exon of *LINC01980* (Figure S2B). Totally, we detected an average of 37.65 LCT events in LUSC (SD = 26.29) and 16.05 events in LUAD samples (SD = 9.56), consistent with previously reported higher L1 activity in LUSC than LUAD [18](Table S1). We compared the overall methylation level (normalized M value as described in Methods) between LUSC and LUAD patients and found LUSC had significantly lower methylation level (Figure S1B), which is consistent with a previous report [20]. We hypothesized that the hypomethylation in LUSC genome might cause L1-ASP activation, which will lead to frequent LCT events. As expected, we also detected significantly increased LCT events and expression in tumor samples compared to normal lung tissues in both LUSC and LUAD (Fig. 1B, Figure S1C, Figure S1D and Table S1). Among LCT events in LUSC, 107 (9.5%) were found in 3’UTR region and 284 (25.1%) in 5’UTR region, while a majority of events (740, 64.5%) were found in coding regions. LUAD has similar distribution (Fig. 1C). Surprisingly the majority of LCTs (67.9% for LUSC, 52.97% for LUAD) occurred at splice-junction sites (less than 3 bp close to the exon boundary), implicating trans-splicing through intronic L1 promoter activation might be a major cause of LCT formation, which was not reported before (Fig. 1C). The LCTs that frequently occurred in at least 3 samples covered many driver genes related to tumorigenicity in both LUSC and LUAD (Fig. 1D and Table S2), including *PON3* [21], *INPP4B* [22, 23], which were also confirmed in TJMUCH LUSC cohort (Figure S1E; Table S3). Interestingly, the functional Gene Ontology enrichment of those genes in recurrent LCT events revealed the significant functions in mitochondrial electron transport and multiple metabolic processes, as well as immune related pathways and WNT pathway (Fig. 1E, Table S4), which were also observed in the TJMUCH LUSC cohort (Figure S1F; Table S5).

### LCT activity is associated with increased metabolic process and suppressed immune response, genomic instability and clinical status

**Fig. 2:**
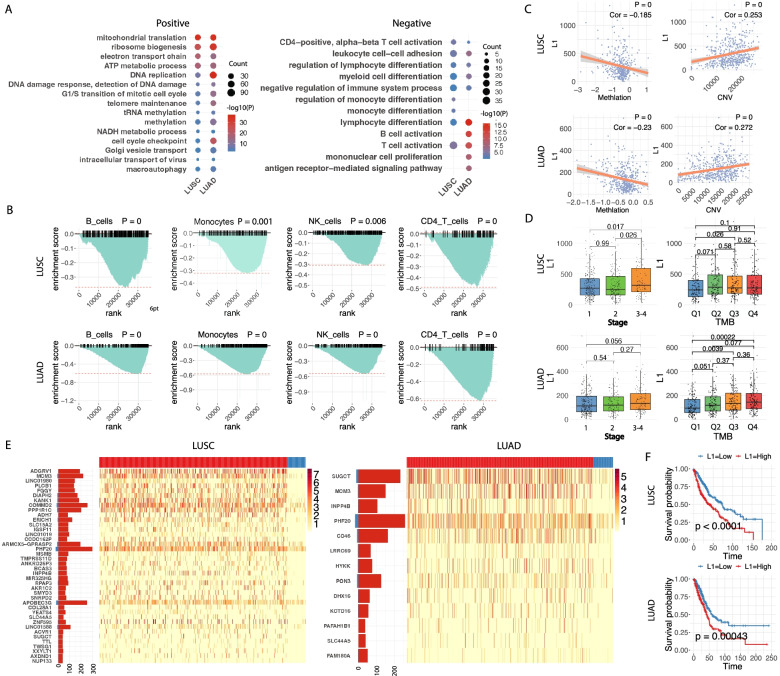
Association of LCT with Metabolic, Immune process, Genomic Instability and Clinical States. **A** Enriched GO terms of genes positively or negatively co-expressed with overall LCT expression in each sample. **B** The GSEA enrichment plot for meta-markers of each immune cell type. Genes are ranked by the fold change of expression in LCT-high samples (left) versus LCT-low samples (right) (**C**) Correlation of methylation level and CNV level with overall LCT activity in each sample. **D** Comparison of LCT expression in different cancer stages and TMB quartiles. **E** Differentially expressed LCT events (named by targeted gene), which are highly expressed in tumor than control. Left panel shows the number of samples containing the LCT event while right panel shows the expression of LCT events among all the samples, grouped by tumor (red) and normal control (blue). **F** Survival curve of patients grouped as L1-high and L1-low by the total expression of survival related LCTs

To determine the association of overall LCT level with genomic, transcriptomic, epigenetic profiles and clinical outcomes, we summarized the reads covering LCTs and normalized by sequencing depth in each tumor sample to represent the overall L1 activity of the corresponding tumor sample. The correlation of L1 activity with RNA sequencing data in both LUSC and LUAD revealed L1 is positively correlated with gene expression of mitochondrial translation and NADH metabolic process, DNA replication/DNA repair and methylation whereas negatively correlated with gene expression in immune response especially T cell immunity, which were also seen in the Tianjin LUSC cohort (Fig. 2A, Figure S3A, Figure S3B and Table S6,7,8,9). Negative correlation of L1 activity with immune response was also confirmed by GSEA analysis with immune cell-type features (Fig. 2B, Figure S3C and Methods). We clearly observed a negative correlation of L1 with methylation levels and positive correlation with genomic instability (copy number aberration data) (Fig. 2C and Methods), as reported earlier [24, 25], consistent with the correlation of L1 integration with the dysregulation of methylation and DNA replication/repair pathways and these transcriptomic, epigenetic and genomic aberrations also promote tumor development and progression [26]. As expected, the L1 activity had positive correlation with tumor stage and TMB level in both LUSC and LUAD (Fig. 2D). These data implicated a role of LCTs in regulating key pathways associated with lung cancer development and progress. These data also indicated that LCTs have potential as a lung cancer diagnostic and prognostic marker.

Next, we performed differential expression analysis between tumor and normal samples in LUSC and LUAD to identify characteristic LCTs associated with each tumor type separately (Methods). We identified 74 differentially expressed LCTs involving 40 genes in LUSC including *L1-ADGRV1*, *L1-LINC01980*, *L1-PLCB1*, and *L1-FGGY* (Fig. 2E, left, Table S10 and Methods), which were also found in TJMUCH cohort (Figure S1G, Table S11 and Methods). In LUAD, 13 differentially expressed LCTs corresponding to 13 genes were discovered (Fig. 2E, right, Table S1^2^ and Methods), among which five events (*L1-MCM3*, *L1-PHF20*, *L1-INPP4B*, *L1-SLC44A5*, *L1-SUGCT*) are overlapped with those detected in LUSC suggesting their common and unified role in carcinogenesis of NSCLC. Interestingly, 2 genes (*INPP4B* and *SUGCT*) are associated with metabolic pathways or disorders [27, 28]. *MCM3* and *INPP4B* has been reported before to be related to cancer [29, 30], while other three might be potential candidates for further study. These differentially expressed LCTs in LUSC and LUAD could serve as a potential diagnostic marker to identify LUSC tumors that are caused by L1 activation. By using a cox regression model, we also identified survival related LCTs in LUSC and LUAD respectively and the summarized expression level of these LCTs could envisioned as possible prognostic markers for NSCLC (Fig. 2F, Table S13-14 and Methods).

### LCTs promote mitochondrial function and metabolic process in tumors at single cell level

**Fig. 3:**
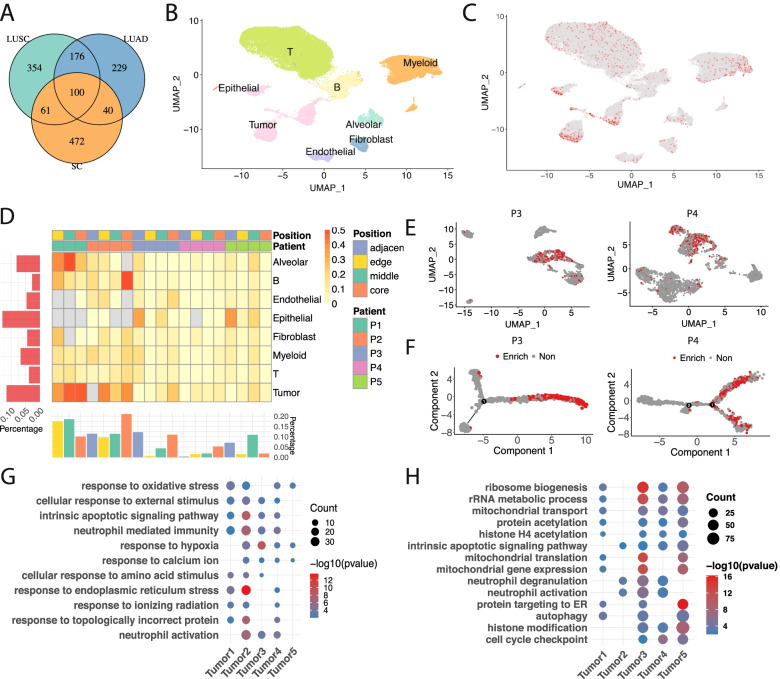
LCT activity at single cell level in NSCLC. **A** The number of overlapped events determined between TCGA bulk RNAseq and single cell sequencing data sets. **B** UMAP plot of cells colored by cell type. **C** UMAP plot of cells colored by LCT activity. Red colored dots represent cells with LCT event detected. **D** The percentage of cells with LCT activity in each cell of each patient. The summary of each patient or each cell type tested is shown as the bar plot on the left and bottom. The UMAP (**E**) plot and pseudo time trajectory analysis (**F**) of tumor cells from patient 3 and patient 4 is shown. Red colored dots represent cells with LCT event detected. **G** Enriched GO termed of genes highly expressed in tumor subtype enriched with LCT events versus other tumor cells. (H) Enriched GO terms of genes highly expressed in LCT positive tumor cells versus LCT negative tumor cells

To investigate LCT events specifically in tumor cell and immune cell in heterogenous cell population in a tumor sample, we applied the ReFuse program to a single cell RNAseq dataset of 19 samples from 5 NSCLC patients including both LUSC and LUAD (Figure S4A and Methods) [31]. Four samples including adjacent healthy tissue, tumor edge, tumor middle and tumor core were collected from 4 patients and one patient donated 3 samples (tumor edge, tumor middle and tumor core). Among the total 19 samples, including tumor edge, tumor middle, tumor core and adjuvant normal tissues, we identified 573 recurrent LCT events, among which a significant portion were detected by bulk RNAseq of TCGA cohort (161 in LUSC, 140 in LUAD and 100 in both) (Fig. 3A, Figure S4A and Table S1^5^). Many highly recurrent LCTs detected by bulk RNAseq data (*L1-FGGY*, *L1-PLCB1*, *L1-ABCB8* and *L1-LINC01980*) were also confirmed in single cell RNAseq dataset. These data demonstrated high consistency of LCT events detected by ReFuse from either bulk or single cell RNAseq data.

To further examine LCT events in each specific cell type in tumor sample, we performed the unsupervised clustering analysis on scRNAseq data and identified major cell types using the markers of NSCLC reported in the original paper and later studies in lung cancer [31, 32] (Fig. 3B and Figure S4C). We then investigated LCT events at single cell level by mapping LCT events to each cell of a specific cell type. Although LCT events are widely distributed among different cell types (Fig. 3C), we found higher LCT events in tumor cells and epithelial cells as demonstrated by a UMAP plot (Fig. 3C) and illustrated from a heatmap (Fig. 3D). We also observed a trend of increasing LCT events from adjacent normal, edge, middle to core in three patients, which might reflect tumor heterogeneity and a trend of increasing tumor cell density from the edge to the core (Fig. 3D).

One interesting finding of LCT events in tumor cells was that, unlike other types of LCT-containing cells which evenly scattered among cell clusters, LCT-containing tumor cells are aggregated in one cluster, implicating distinct transcriptomic features of LCT-enriched tumor cells different from the rest of tumor cells. We further extracted tumor cells from each patient and conducted subtype analysis (Methods). We surprisingly found an individual tumor cell cluster highly enriched with LCT events (Fig. 3E; Figure S4) amongst all the patients analyzed. When aligning those cell populations along pseudo time trajectory (Methods), we found the LCT-containing tumor cell cluster concentrated on one end of the trajectory (Fig. 3F; Figure S5A), suggesting their unique cell fate within in tumor cells as shown. Differential expressed gene (DEG) analysis and GO enrichment indicated that in the most differentially expressed genes in LCT-enriched tumor cell cluster (Figure S5Band Table S16) related to response to stress, such as response to oxidative stress, cellular response to external stimulus, and response to hypoxia in multiple patients (Fig. 3G and Table S17). In a summary, the LCT-enriched NSCLC tumor cell sub-cluster represents a specific tumor cell status with increased stress response.

To further test the influence of LCT on transcriptomic dysregulation at a single cell level, we performed DEG analysis between LCT-positive and LCT-negative cells in the LCT-enriched tumor sub-cluster from five patients (Figure S5Cand Table S1^8^). GO enrichment identified increased mitochondrial function, ribosome biogenesis and activated metabolic pathway in LCT-positive tumor cells, which is consistent with the result from bulk RNAseq analysis presented above (Fig. 3H and Table S19). This LCT activity analysis from scRNASeq analysis indicate that the L1 plays an oncogenic role by intronic L1 promoter activation in a cancer gene, leading to the formation of LCT, involved with mitochondrial function and metabolic process, thereby, reprogramming metabolism favorable for tumorigenicity.

### *L1-FGGY* initiated arachidonic acid (AA) metabolism reprogramming and activated 12-LOX pathway in LUSC

**Fig. 4:**
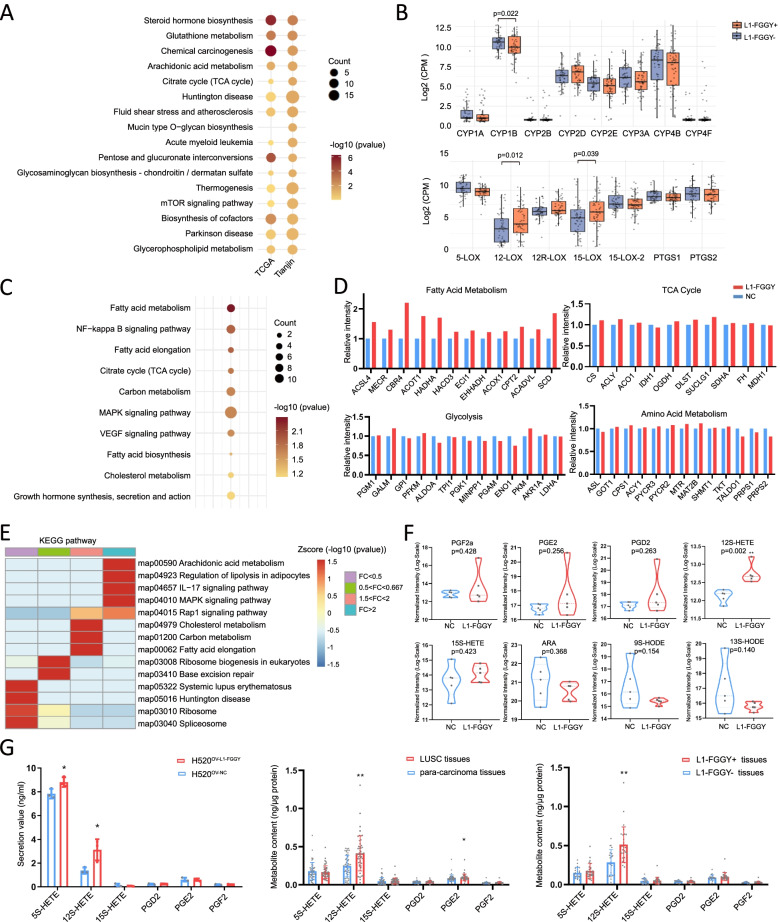
*L1-FGGY* initiated arachidonic acid (AA) metabolism reprogramming and activated 12-LOX pathway in LUSC. **A** The KEGG pathway enrichment analysis of differential expression between *L1-FGGY*^+^ tissues and *L1-FGGY*^−^ tissues based on RNAseq data from TCGA and an independent Chinese cohort of 109 LUSC tumor samples (52 *L1-FGGY*^+^ vs. 57 *L1-FGGY*^−^) from TJMUCH. **B** The gene expression profiles (log_2_ (count per million (CPM))) of the downstream key components involved in AA metabolism was compared between *L1-FGGY*^+^ tissues and *L1-FGGY*^−^ tissues based on the RNAseq data. **C** The KEGG pathway enrichment analysis of differential expression between H520^OV−*L1−FGGY*^ and H520^OV−CTRL^ based on proteomics analysis. **D** The protein expression involved in different metabolic pathways was compared between H520^OV−*L1−FGGY*^ and H520^OV−CTRL^ based on proteomics analysis. **E** The KEGG pathway enrichment analysis of proteins with different fold changes between H520^OV−*L1−FGGY*^ and H520^OV−CTRL^ based on proteomics analysis. **F** The results of metabolomics assay targeting AA pathway metabolites were compared between H520^OV−*L1−FGGY*^ and H520^OV−CTRL^. **G** The ELISA results of AA pathway metabolites from cells and tissues. The data are shown in plots. * and ** indicate *p* < 0.05 and *p* < 0.01, respectively between the groups as indicated

Considering the high frequency and potential biological functions of *L1-FGGY* in metabolic control mechanisms, here we selected it for further explorations to elucidate the underlying oncogenic role of L1 in tumorigenesis and tumor progression. The comparison of expression profiles between *L1-FGGY*^+^ and *L1-FGGY*^−^ samples from TCGA LUSC specimens identified altered control of metabolic pathways, including glutathione metabolism, AA metabolism, and drug metabolism (Fig. 4A), which was further confirmed from an independent Chinese cohort of 109 LUSC tumor samples (52 *L1-FGGY*^+^ vs. 57 *L1-FGGY*^−^) (Fig. 4A). We next examined the expression levels of the specific genes involved in the downstream key pathways of AA metabolism (i.e. cytochrome P450 (CYP450), lipoxygenase (LOX), and cyclooxygenase (COX)) [33] and observed 12-LOX and 15-LOX (both in LOX pathway) were elevated while other pathways were either downregulated or reflected no significant change (Fig. 4B), implicating *L1-FGGY* to activate the LOX pathway through AA metabolism.

In order to further investigate the biological processes affected by *L1-FGGY*, we constructed NCI-H520 cells over-expressing *L1-FGGY* (H520^OV−*L1−FGGY*^), and empty vector (H520^OV−CTRL^). Proteomics analysis of these two cell lines identified 951 (618 up and 333 down) dysregulated proteins in H520^OV−*L1−FGGY*^ at an adjusted *p* value of 0.05 with enriched functions in fatty acid metabolism (Fig. 4C and 4D), particularly proteins with more than twofold increase in H520^OV−*L1−FGGY*^ were enriched in AA metabolism pathway, regulation of lipolysis in adipocytes, IL-17 signaling pathway and MAPK signaling pathway (Fig. 4E). We also observed *L1-FGGY* altered the morphology of mitochondria slightly, significantly promoted mitochondrial oxidative phosphorylation activity, membrane enhancement and ATP production (Figure S6). Further metabolomics analysis targeting AA pathway analytes detected increased level of 12S-HETE (a downstream metabolite of 12-LOX), but not 15S-HETE (a downstream metabolite of 15-LOX) in H520^OV−*L1−FGGY*^ (Fig. 4F). We then measured the levels of AA metabolites using Enzyme-linked immunosorbent assay (ELISA) kits. The increased secretion levels of 12S-HETE was detected in H520^OV−*L1−FGGY*^ vs H520^OV−CTRL^ cell lines (Fig. 4G) and further validated in 50 LUSC tissues compared to matched adjacent normal tissues (Table S20-S21) as well as *L1-FGGY*^+^ vs *FGGY*^−^ tumor tissues (Fig. 4G).

Collectively, these results indicated that *L1-FGGY* corresponds with induced lipid metabolism, specifically reprogramming of AA metabolism, which activates the downstream 12-LOX pathway, leading to an increased production of the 12S-HETE metabolite.

### *L1-FGGY* exhibited an oncogenic role dependent on activation of 12-LOX/GPR31 metabolic pathway

**Fig. 5:**
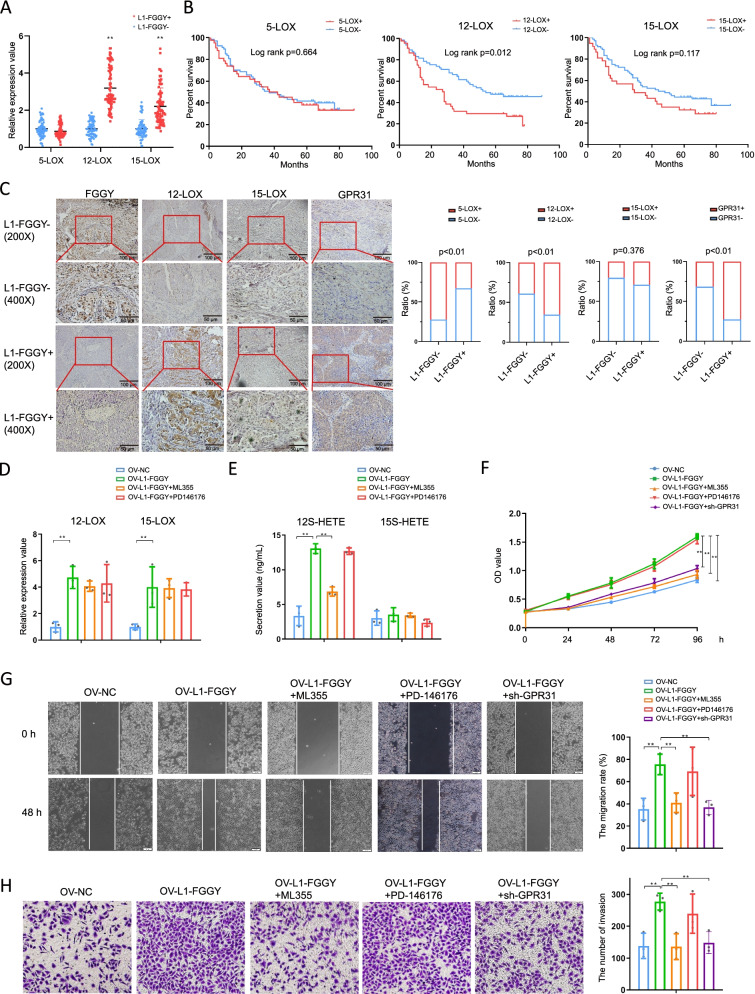
*L1-FGGY* exhibited an oncogenic role dependent on activation of 12-LOX/GPR31 metabolic pathway. **A** The relative RNA expression values of 5-LOX, 12-LOX and 15-LOX detected in 147 LUSC tumor samples (77 *L1-FGGY*^+^ vs. 70 *L1-FGGY*^−^) from TJMUCH. **B** The OS was compared between 5-LOX^+^ and 5-LOX^−^, 12-LOX^+^ and 12-LOX^−^, 15-LOX^+^ and 15-LOX^−^ patients respectively grouped according to mRNA levels. **C** Different resolutions of IHC staining results of the FGGY, 12-LOX, 15-LOX and GPR31 antigen expression in *L1-FGGY*^−^ and *L1-FGGY*^+^ LUSC tumor samples. The statistical results of IHC staining were shown at right. **D** The relative RNA expression of 12-LOX and 15-LOX detected in H520^OV−CTRL^ and H520^OV−*L1−FGGY*^, as well as in H520^OV−*L1−FGGY*^ treated with either ML355 or PD146176. **E** The secretion value of 12S-HETE and 15S-HETE detected in H520^OV−CTRL^, H520^OV−*L1−FGGY*^, and H520^OV−*L1−FGGY*^ treated with either ML355 or PD146176. **F** The proliferation of H520^OV−CTRL^, H520^OV−*L1−FGGY*^, H520^OV−*L1−FGGY*+sh−*GPR31*^, and H520^OV−*L1−FGGY*^ treated with either ML355 or PD146176 was detected using CCK8 method. **G** Representative images of H520^OV−CTRL^, H520^OV−*L1−FGGY*^, H520^OV−*L1−FGGY*+sh−*GPR31*^, and H520^OV−*L1−FGGY*^ treated with either ML355 or PD146176 in wound healing assays. The statistical results of migration rate were shown at the right of the panel. (H) Representative images of H520^OV−CTRL^, H520^OV−*L1−FGGY*^, H520^OV−*L1−FGGY*+sh−*GPR31*^, and H520^OV−*L1−FGGY*^ treated with either ML355 or PD146176 in trans-well invasion assays. The statistical results of invasion number were shown at right. The data are shown as mean ± SD with plots. * and ** indicate *p* < 0.05 and *p* < 0.01, respectively between the groups as indicated

We further explored the three central enzymes involved in the LOX metabolic pathways, i.e. 5-LOX, 12-LOX, and 15-LOX [34] in 147 LUSC tumor samples (77 *L1-FGGY*^+^ vs. 70 *L1-FGGY*^−^) from the TJMUCH cohort (Table S20-S21). We observed increased expression of both 12-LOX and 15-LOX rather than 5-LOX in *L1-FGGY*^+^ tissues compared to *L1-FGGY*^−^ tissues (Fig. 5A) and the increased expression of 12-LOX was exclusively associated with poor survival (Fig. 5B). At the protein level, a high level 12-LOX (but not 15-LOX) IHC staining was observed in *L1-FGGY*^+^ tumor tissues (Fig. 5C). GPR31, the membrane receptor of 12-LOX metabolic 12S-HETE [35] was upregulated in *L1-FGGY*^+^ tissues compared to *L1-FGGY*^−^ tissues (Fig. 5C). In order to determine whether *L1-FGGY* induced tumorigenicity depends on 12-LOX or 15-LOX metabolic pathway, we treated H520^OV−*L1−FGGY*^ cells with either 12-LOX inhibitor ML355 [36] or 15-LOX inhibitor PD146176 [37]. We found that over-expression of *L1-FGGY* caused upregulation of 12-LOX and 15-LOX gene expression, but only an increased release of 12S-HETE but not of 15S-HETE (Fig. 5D-E). As expected, ML355 (but not PD146176) efficiently suppressed the increased 12S-HETE release in H520^OV−*L1−FGGY*^ cells though both inhibitors had no effect on 15S-HETE level (Fig. 5E). We further explored the role of 12-LOX in *L1-FGGY*-driven tumorigenicity. We found an accelerated proliferation rate in H520^OV−*L1−FGGY*^ which was significantly reduced by ML355 treatment (Fig. 5F). Next, we found that the wound closure rate (WCR) of H520^OV−*L1−FGGY*^ was significantly higher than H520^OV−CTRL^ (Fig. 5G), Consistently, more of those cells expressing *L1-FGGY* migrated across the matrigel layer after 48 h (Fig. 5H), whereby, the increased migration and invasion abilities in H520^OV−*L1−FGGY*^ were both reversed by ML355 treatment, but not with the treatment of PD146176 (Fig. 5G-H). We further confirmed our findings in a different LUSC cell line, SK-MES-1, in which we previously confirmed *L1-FGGY* promoted cell proliferation and migration, reduced cell apoptosis, as well as promoted AA metabolism [19]. Figure S7 demonstrated that, in SK-MES-1 cell, over-expression of *L1-FGGY* caused upregulation of 12-LOX and metabolite 12S-HETE and thus promoted cell proliferation and migration dependent on activating the 12-LOX/GPR31 metabolic pathway.

Taken together, these results suggest that *L1-FGGY* exhibits an oncogenic role by activating the metabolic pathway that exploits 12-LOX which can be partially reversed by ML355.

### *L1-FGGY* activated Wnt signaling pathway in LUSC via accelerating GPR31 deubiquitination and enhancing 12S-HETE/GPR31 interaction

**Fig. 6:**
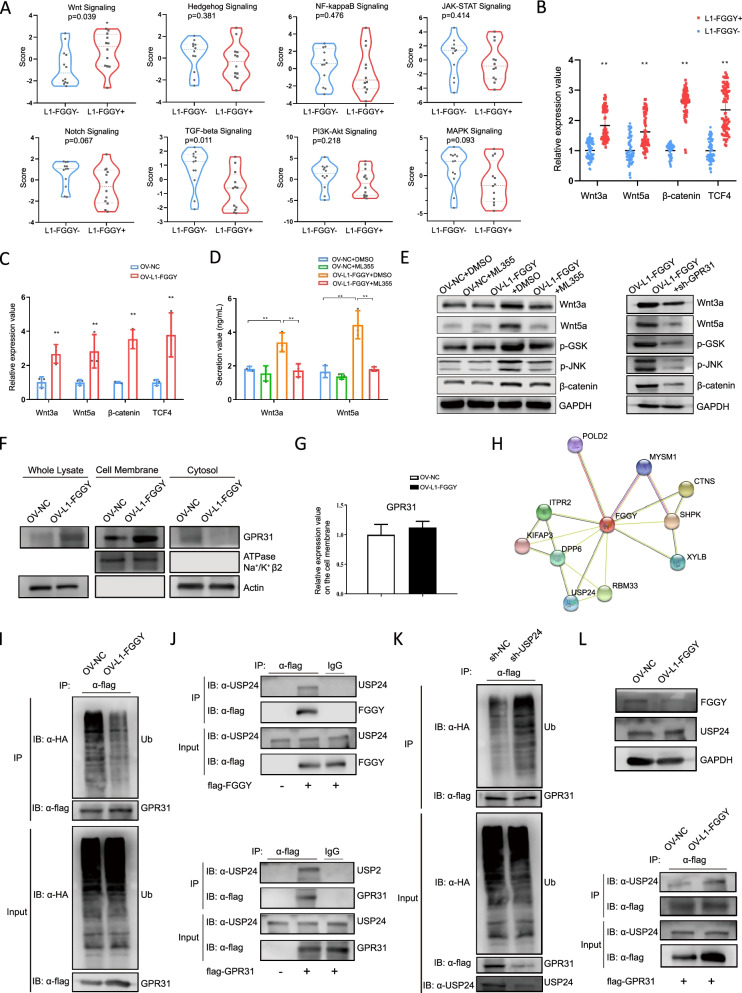
*L1-FGGY* activated Wnt signaling pathway in LUSC via accelerating GPR31 deubiquitination and enhancing 12S-HETE/GPR31 interaction. **A** The pathway scores of different signaling pathways were compared between *L1-FGGY*^+^ tissues and *L1-FGGY*^−^ tissues (*n* = 12) detected in nCounter® PanCancer IO-360™ Panel. **B** The relative RNA expression of Wnt3a, Wnt5a, β-catenin and TCF4 detected in 147 LUSC tumor samples from TJMUCH as mentioned above. **C** The relative RNA expression of Wnt3a, Wnt5a, β-catenin and TCF4 detected in H520^OV−CTRL^ and H520^OV−*L1−FGGY*^. **D** The secretion value of Wnt3a and Wnt5a detected in H520^OV−CTRL^ and H520^OV−*L1−FGGY*^ treated with ML355. The cells treated with DMSO were as controls. **E** Western blot results to detect protein expression of Wnt3a, Wnt5a, β-catenin, and the phosphorylation level of GSK and JNK in H520^OV−CTRL^ and H520^OV−*L1−FGGY*^ treated with either DMSO or ML355 were shown at left. And western blot results to detect key proteins involved in Wnt signaling pathway in H520^OV−*L1−FGGY*^ and H520^OV−*L1−FGGY*+sh−*GPR31*^ were shown at right. **F** Western blot results to detect expression of GPR31 from whole lysate, fraction of cell membrane and cytosol individually in H520^OV−CTRL^ and H520^OV−*L1−FGGY*^. **G** The relative RNA expression of GPR31 detected in H520^OV−CTRL^ and H520^OV−*L1−FGGY*^. **H** FGGY is indicated to have an association with USP24 by STRING. **I** The cell lysates from H520^OV−CTRL^ and H520^OV−*L1−FGGY*^ were used in immunoprecipitation (IP) with flag antibody to detect the ubiquitination level of flag-GPR31. MG132 (10 μM) was added for 6 h before the cells were harvested. **J** Interaction of FGGY with USP24 (top) and interaction of GPR31 with USP24 (bottom). H520 cells were transfected with the plasmids as indicated. At 24 h post-transfection, the cell lysates were used in IP and immunoblotting (IB) using the antibodies as indicated. **K** The cell lysates from H520^sh−CTRL^ and H520^sh−*USP24*^ were used in IP with flag antibody to detect the ubiquitination level of flag-GPR31. MG132 (10 μM) was added for 6 h before the cells were harvested. **L** Western blot results to detect protein expression of FGGY and USP24 in H520^OV−CTRL^ and H520^OV−*L1−FGGY*^ were shown on top. IP results to detect protein level of USP24 which has an interaction with flag-GPR31 in H520^OV−CTRL^ and H520^OV−*L1−FGGY*^ were shown below

Next, in order to explore which signaling pathways are involved in *L1-FGGY*-driven carcinogenesis, we profiled 12 pairs of *L1-FGGY*^+^/*L1-FGGY*^−^ LUSC tissues with nCounter® PanCancer IO-360™ Panel [38]. We observed a trend by the elevation of cell proliferation and metabolic stress, the loss of apoptosis and autophagy in *L1-FGGY*^+^ tissues (Figure S8A). Specially, a significant upregulation of Wnt signaling pathway was detected in *L1-FGGY*^+^ tissues compared to *L1-FGGY*^−^ tissues (Fig. 6A). Our data also showed a significant loss of several immune-related cell signaling pathways (i.e., antigen presentation, immune cell adhesion and migration, interferon signaling, and cytokine and chemokine signaling) (Figure S8A), and cell signatures (tumor infiltrating lymphocyte (TILs), dendritic cells (DCs) and mast cells) (Figure S8B) in *L1-FGGY*^+^ tissues, indicating *L1-FGGY* corresponded with a dysregulated immune microenvironment.

We next validated the upregulation of Wnt signaling in the 147 LUSC tumor samples by qPCR and the results showed that the expression of key components in Wnt signaling, i.e., Wnt3a, Wnt5a, β-catenin, and TCF4 were all significantly higher in *L1-FGGY*^+^ tissues than *L1-FGGY*^−^ tissues (Fig. 6B), which were then validated in the H520^OV−*L1−FGGY*^ cell line (Fig. 6C). Furthermore, the over expression of Wnt3a and Wnt5a was suppressed by the 12-LOX inhibitor ML355 (Fig. 6D). The protein expression of Wnt3a, Wnt5a, β-catenin, and the phosphorylation level of GSK and JNK were higher in H520^OV−*L1−FGGY*^ (Fig. 6E). And either inhibition of 12-LOX or suppressing *GPR31* could block the upregulation of Wnt signaling in H520^OV−*L1−FGGY*^ (Fig. 6E). These findings reveal that *L1-FGGY* activated Wnt signaling depends on expression of the 12-LOX/GPR31 metabolic pathway.

Since 12S-HETE/GPR31 interaction is the key component of 12-LOX/GPR31 metabolic pathway, we also examined the expression and distribution of GPR31 protein in H520^OV−*L1−FGGY*^ cells. We found *L1-FGGY* induced a high level of GPR31 protein in the cell membrane rather than cytosol in H520^OV−*L1−FGGY*^ cell (Fig. 6F). Considering no significant change in RNA level of GPR31 by *L1-FGGY* overexpression (Fig. 6G), the increased GPR31 protein expression induced by *L1-FGGY* led us to explore the possibility that ubiquitin-mediated degradation of the protein is altered by *L1-FGGY*, as the FGGY protein was shown to have an association with USP24, a ubiquitin carboxyl-terminal hydrolase, by STRING analysis, as a protein–protein interaction network prediction tool (Fig. 6H). We firstly observed that the ubiquitination level of GPR31 was reduced in H520^OV−*L1−FGGY*^ (Fig. 6I). Then we detected binding of USP24 with FGGY or GPR31 (Fig. 6J), illustrating the physical interaction of USP24 with either FGGY or GPR31, whereby, the ubiquitination of GPR31 was increased by USP24 knockdown (Fig. 6K) to indicate that USP24 acts as a deubiquitination enzyme of GPR31. Considering that *L1-FGGY* limits FGGY expression and that FGGY interacts with USP24, a de-ubiquitination enzyme of GPR31, we consider that *L1-FGGY-*mediated decrease of *FGGY* abundance may free USP24 to interact with GPR31, leading to GPR31 de-ubiquitination. The interaction of GPR31 with USP24 was increased in H520^OV−*L1−FGGY*^ cells (Fig. 6L) by using an immunoprecipitation assay, whereas, the total USP24 protein level in cell lysates did not change. This result appears consistent with our hypothesis.

Collectively, these findings implied that the *L1-FGGY* reduces the expression of FGGY, thereby, facilitating the binding of more USP24 to GPR31 that increases GPR31 de-ubiquitination and activation of metabolic pathway for AA/12-LOX/12S-HETE and the subsequent signaling through the Wnt pathway in tumor cells for enhanced cell proliferation and invasion (Figure S9).

### Reverse transcriptase inhibitor and 12-LOX inhibitor impaired growth of LUSC xenografts and reversed immunosuppressive microenvironment in vivo

**Fig. 7:**
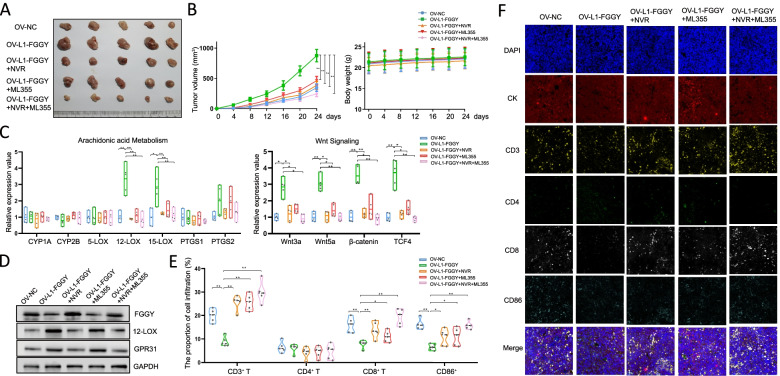
Reverse transcriptase inhibitor and 12-LOX inhibitor impaired growth of LUSC xenografts and reversed immunosuppressive microenvironment in vivo. **A** KLN205^OV−CTRL^ and KLN205^OV−*L1−FGGY*^ were inoculated subcutaneously in the DBA2 mice which were subjected to either NVR (50 mg/kg/day) or ML355 (3 mg/kg/day) treatment, or subjected with the two inhibitors simultaneously. Representative images of the forming tumors were shown. **B** The tumor volume (left panel) and body weight (right panel) of the mice at various time points upon injection. **C** The qPCR results of genes involved in AA metabolic pathways (left) and qPCR results of genes involved in Wnt signaling (right) were shown as relative expression values in KLN205^OV−CTRL^ mouse group, KLN205^OV−*L1−FGGY*^ mouse group, NVR-treated KLN205^OV−*L1−FGGY*^ mice, ML355-treated KLN205^OV−*L1−FGGY*^ mice and NVR + ML355-treated KLN205^OV−*L1−FGGY*^ mouse group. **D** Western blot results to detect expression of FGGY, 12-LOX, and GPR31 in different mouse groups we constructed. **E** The proportions of various immunocytes in different mouse groups using multispectral immuno-fluorescent staining. **F** Representative images of multispectral immuno-fluorescent staining results

We tested the roles of the 12-LOX metabolic pathway activated by *L1-FGGY* for tumorigenesis in vivo by over expressing *L1-FGGY* in the mouse LUSC cell line KLN205 (KLN205^OV−*L1−FGGY*^). The KLN205^OV−CTRL^ and KLN205^OV−*L1−FGGY*^ cells were engrafted subcutaneously in DBA2 mice as xenografts, followed by administration of either NVR (50 mg/kg/day) or ML355 (3 mg/kg/day), or combined. After 24 days, the average volume of the tumors analyzed in mouse group receiving the KLN205^OV−*L1−FGGY*^ cells were characterized as being much larger than those in KLN205^OV−CTRL^ group (*p* < 0.05, Fig. 7A-B). Furthermore, the growth of the xenografts we generated within KLN205^OV−*L1−FGGY*^ cell group were significantly reduced by either NVR or ML355 treatment, and further reduced when treated with the two inhibitors, simultaneously (Fig. 7A-B). The body weight curves indicated that both inhibitors displayed comparable physiological responses since neither whole-body weight lost, food intake or mortality occurred during the treatment (Fig. 7B).

Consistent with the results from human cell lines used, the key enzymes involved in fatty acid metabolism were elevated in the mouse group receiving the KLN205^OV−*L1−FGGY*^ cells, which could be inhibited by either NVR or ML355 treatment (Figure S10A). As anticipated, the transcription of the enzymes for 12-LOX and 15-LOX for AA metabolism pathway was elevated in the mouse group represented as KLN205^OV−*L1−FGGY*^ and was reduced following the treatment with the drug inhibitors (Fig. 7C). Similarly, the mRNA level of Wnt3a, Wnt5a, β-catenin, and TCF4 was all significantly higher in the KLN205^OV−*L1−FGGY*^ group and was reduced after inhibitor treatment by either drug (Fig. 7C). Consistent with the results in human LUSC cells, significant decrease of FGGY protein was detected in KLN205^OV−*L1−FGGY*^ mice which was fully recovered by NVR and combined treatment, but partly recovered by ML355 treatment. On the contrast, increased protein level of 12-LOX and GPR31 was detected in KLN205^OV−*L1−FGGY*^ mice which was fully suppressed by NVR and combined treatment, but partly suppressed by ML355 alone (Fig. 7D).

Tumor development is always accompanied with the changes in the immune microenvironment [39]. We then further examined the proportions of various immune cell populations from different mouse groups by using multispectral immuno-fluorescent staining. Reduced CD3^+^ T cells and CD8^+^ T cells were observed in KLN205^OV−*L1−FGGY*^ mouse group, indicating *L1-FGGY* affected T lymphocyte populations, especially those involving cytotoxic T cell infiltration, thereby, promoted an immunosuppressive or exhaustive tumor microenvironment. Similarly, the number of infiltrating CD86^+^ cells, which is a biomarker for DCs [40], was dramatically reduced in the mouse group KLN205^OV−*L1−FGGY*^ (Fig. 7E-F), consistent with the results of cell type analysis in human tumor tissues by IO360. As we expected, the decrease of these immune cell populations from the KLN205^OV−*L1−FGGY*^ mouse group was reversed by the treatment with two inhibitors (Fig. 7E-F). The similar distribution pattern of these immunocytes was obtained by flow cytometry (Figure S10B).

### Precision-guided approaches for lung cancer patients with a poor prognosis that correspond with high LCT activity

**Fig. 8:**
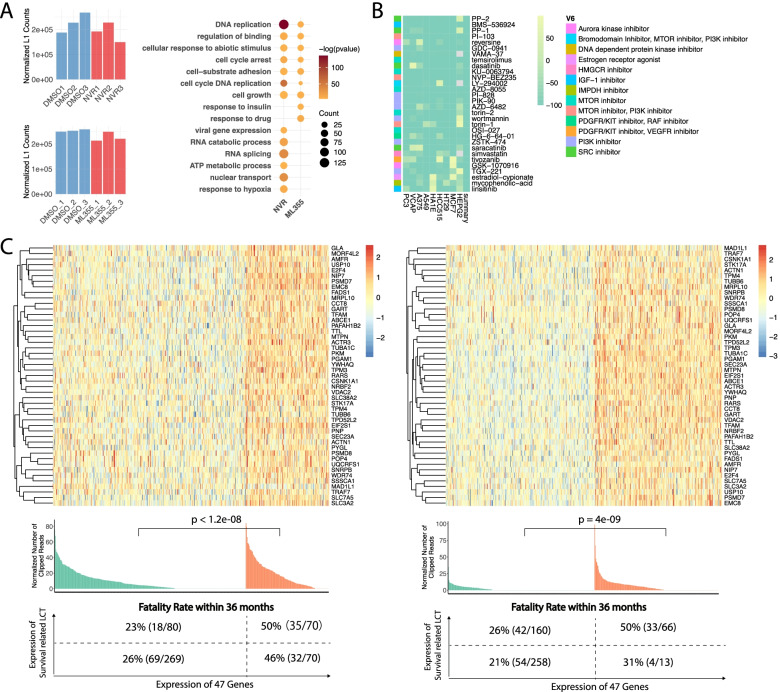
Precision-guided evidence for lung cancer patients with a poor prognosis that correspond with high LCT activity. **A** Overall L1 expression in samples treated with NVR or ML355 are illustrated. L1 expression was normalized as read per million for each sample where the enriched GO terms of those genes whose expression decreased after treatment is noted. **B** The top drug candidates for the 46 LCT related genes. Heatmap shows the correlation score of each drug in each cell line predicted using the “Cmap” database. **C** Top panel shows the z-normalized expression of the 46 genes in all tumor samples. Middle panel shows the overall expression of LCT in two groups based on the expression of 46 genes. On the lower panel, two groups are divided into four based on the total expression value of survival related LCT in each sample. The mortality rate and number of mortality cases within 36 months of each group was labeled in each group

By summarizing the reads that mapped to L1 in RNA sequencing data of the H520^OV−*L1−FGGY*^ cell line treated with NVR or ML355, we observed a decrease of L1 activity after the treatment with either inhibitor (Fig. 8A and Methods), The reduction of expression from those gene transcripts following NVR and ML355 treatment were enriched in ATP metabolic process, cell cycle, cell growth, which are associated with LCT activity (Fig. 8A). These data along with their capacity to inhibit tumor growth implicate a potential application of NVR and ML355 or other anti-HIV and anti-metabolic drugs in treating lung cancer patients with a high level of LCT activity and poor survival outcome.

To best stratify which lung cancer patients who would benefit with anti-HIV and anti-metabolic drugs, we determined 47 genes whose expression is positively-correlated with survival-related LCT activity in bulk RNA sequencing data and increased in LCT-positive vs LCT-negative tumor cells from single cell sequencing data (Methods; Table S22). By performing drug repurposing on these 47 genes with CMAP database, we identified a set of drugs including PI3K/MTOR/HMGCR inhibitors which could be repurposed to suppress the expression of these 47 genes (Fig. 8B). Simvastatin is one of these predicted HMGCR inhibitors and widely used to lower cholesterol level. We found repurposed drug and other metabolic drug (Metaformin) can suppress L1 activity and recover metabolism reprogramming in cancer cell lines by reducing expression of multiple molecules in mitochondria and metabolic processes in public datasets (Figure S11A-C), suggesting these anti-metabolic drugs along with anti-HIV drugs could be considered as potential therapies given the poor prognosis of lung cancer patients with a high LCT activity. Based on overall expression of these 47 candidate genes and survival-associated LCTs, we stratified the LUSC and LUAD samples from TCGA into four groups (Fig. 8C). The thresholds were optimized based on the 36 months fatality rate of patients in each group (Methods). The patient group with the high expression of 47 genes and LCTs showed over 50% rate in mortality within 3 years, suggesting these could be candidates for consideration with these drugs, especially when other therapies fail.

## Discussion

In this study, we developed a local-alignment based bioinformatic tool to detect genome-wide LCTs with a high sensitivity in lung cancer in both bulk and single cell RNA sequencing data. We observe that LCTs are preferably involved among genes with mitochondrial function(s) and metabolic process, leading to the reprogramming of metabolism favorable for tumor cell growth and survival and subsequent progression leading to metastasis. In particular, in our comprehensive in vitro and in vivo functional studies of the LCT to reveal the highly recurrent *L1-FGGY* that directly influences oncogenesis by stimulating the 12-LOX pathway through the pathways that manage arachidonic acid and fatty acid metabolism which in-turn stimulates Wnt signaling and other oncogenic signaling pathways. Finally, a set of metabolic transcripts and LCT markers were determined to identify the patients with a high risk of poor outcomes who would benefit by treatments with NVR and other inhibitors of metabolism. Our investigations are initial studies using genome-wide and accurate detection of LCTs with high sensitivity using both bulk and single cell RNA sequencing data from lung cancer specimens and represents to reveal the functional role of L1 integration in metabolic programming in tumorigenicity of lung cancer. This could reflect the similar behavior of LCTs in other cancer types.

In previous study, we used the DeFuse program that was used to detect the fusion from two different gene loci, to identify a limited number of LCTs for LUSC. Considering this general approach may not detect many fusion events within a gene locus, we developed the ReFuse program, which can identify L1-chimeric transcript using a more sensitive detection methods and alignments of the genomic locations of the L1 position and LCT partner. Here, we have identified many LCTs with the majority involving splice-junction sites through L1-ASP activation that implicate intronic L1-ASP activation as a predominant mechanism of how L1 integration is involved in lung cancer tumorigenicity at the transcript level. However, due to the high similarity of short-read sequences of L1 families, there are limitations is revealing the exact origin of many L1s from short read RNA sequencing, therefore, long read sequencing (ISO-Seq) of the whole LCT could enhance the analysis as a framework and as a reference transcriptome for considering the origins of L1s to target their LCTs and further validate their frequencies and importance in human cancer.

Metabolic reprogramming has been shown as a means of cancer cells to navigate a microenvironment favorable to promote tumor occurrence and progression [41, 42]. Specific metabolites can directly participate in the transformation processes or support the biological processes that facilitate tumor growth and progression [43, 44] by a variety of internal and external factors. The downstream pathways of oncogenes and tumor suppressors have been reported to regulate the metabolism of cancer cells [45]. Genomic changes may also lead to an increase or decrease in the expression of genes encoding metabolic enzymes [46, 47]. As a widely distributed transposable element, L1 has been known as promoting tumor development by disturbing the transcription of tumor-related genes, as well as trigging genome instability, however, the roles of L1 in directing tumor cell metabolism and the surrounding microenvironment remains unclear. In this LCT study, we observed the L1s form LCT transcripts by preferably integrating with metabolic genes to affect mitochondrial function and metabolic process, which indicated a promising mechanism in cancer metabolism modulation, and even put forward a novel mechanism of L1 regulation in cancer development.

The balance of glucose and lipid metabolism is important for maintaining cell physiological homeostasis and normal biological functions [48, 49]. Dysfunction of glucose and lipid metabolism can lead to a variety of diseases, especially in cancer [50]. In LUSC, there is an obvious imbalance of glucose and lipid metabolism, which presents upregulation of fatty acid oxidation [51, 52]. Targeted inhibition of fatty acid synthesis can effectively reduce tumor cell proliferation and invasion [42], suggesting that lipid reprogramming is of great significance for maintaining the progression of LUSC. Therefore, breaking through the traditional therapy and mining and potential therapeutic targets at the metabolome level might improve the prognosis and outcome of LUSC.

*FGGY* is a metabolic gene which encodes carbohydrate kinase [53]. *FGGY* was first reported to be related with sporadic amyotrophic lateral sclerosis [54]. Besides that, FGGY can regulate dietary obesity in mice by regulating lipid metabolism [55]. Here we showed that there was an abnormal fatty acid metabolism in *L1-FGGY*^+^ LUSC. Specially, *L1-FGGY* activated the downstream of AA metabolism, i.e. 12-LOX/GPR31/Wnt signaling. AA is an essential fatty acid, and its metabolites participate in a variety of physiological activities in cells, such as cell proliferation, migration, and apoptosis [56]. AA metabolism includes three pathways: LOX, CYP450 and COX [57]. Among them, LOX is a key enzyme in the metabolism of fatty acid, which catalyzes AA to generate HETE and other biologically active metabolites, which could affect cell metabolism and signal transduction, thereby playing an important role in cancer cells and inflammatory response [58]. Fatty acids could promote the expansion of natural killer cells by improving energy metabolism, including enhancing the oxygen consumption rate (OCR), promoting ATP production and elevating the energy flux [20]. PGE2, an AA metabolism, has been reported to enhance oxidative phosphorylation in macrophages [59]. Here consistent with the analysis results from database which indicated the LCT transcripts affected mitochondrial function, we observed *L1-FGGY* could promote mitochondrial oxidative phosphorylation activity, enhance membrane potential and produce more ATP levels. We noticed the increased degree of oxidative phosphorylation triggered by *L1-FGGY* alone was modest, which indicated *L1-FGGY* and other LCT transcripts also co-played roles in mitochondrial function alteration and thus metabolic reprogramming. Since the results from transmission electron microscopy (TEM) showed *L1-FGGY* did not alter the morphology of mitochondria severely, we hypothesized that the accelerated energy metabolism and more energy production might be caused by the activated AA metabolism, but not the alteration of mitochondrial morphology.

GPR31 is a member of the G protein-coupled receptor (GPCR) family and is located in the cell membrane [35, 60]. A large amount of evidence shows that GPCR is important for 12S-HETE-mediated signal transduction and may be involved in the progression of a variety of tumors [35]. We found that *L1-FGGY* increased the expression of GPR31 on the cell membrane, but did not affect its RNA level, suggesting *L1-FGGY* may regulate the membrane expression of GPR31 through post-translational modification. It is reported in the literature that the level of GPCR membrane protein is regulated by deubiquitinating enzyme (USP) [61]. USP regulates the ubiquitination level of the target protein GPCR to reduce GPCR degradation and increase its expression on the cell membrane [62]. Here we discovered that *L1-FGGY* down-regulated the expression of *FGGY*, followed by reducing the binding of FGGY and USP24, which increased free USP24 to increase the deubiquitination level of GPR31, and eventually promoted its membrane expression and therefore activated 12-LOX/Wnt signaling.

The coordination between the immune, stromal and tumor cell populations within the microenvironment play a central role in navigating tumorigenicity and metastasis [39]. Our study indicates that 12S-HETE, the 12-LOX metabolite, was involved in *L1-FGGY* mediated AA pathway activation and immune microenvironment dysregulation. 12S-HETE has previously been identified as a mediator in inflammatory response [63, 64]. In hepatocytes, treatment with 12S-HETE resulted in greater p65 (RELA), JNK, p38 and ERK phosphorylation and inflammatory gene expression, suggesting the proinflammatory action of 12S-HETE [64]. While delivery of 12S-HETE to the airway of mice has been reported to attenuate allergic airway inflammation [65]. Furthermore, 12S-HETE has been shown to promote secretion of IL-4 and IL-13, thereby polarizing macrophages to a more M2-like phenotype [66]. In this study, we observed the *L1-FGGY* directed elevation of 12S-HETE also involves proteins with functions in T cell activation and L1 activity is correlated with the down-regulation of T cell activation pathways, implicating a negative regulation of L1 in tumor immune microenvironment as an alternative mechanism for the tumor to escape from immune surveillance, and even suggesting a novel treatment by combined of targeting L1 and immunotherapy. However, more extensive functional and mechanism-based studies with immune cells are needed to elucidate the roles of L1 in innate and adaptive immune response activity associated with tumorigenicity and progression.

Here we showed L1 promoted tumor progression via coordinating its effects on multiple metabolic processes and immune activities. We not only proposed L1 as a candidate marker for cancer diagnosis and prognosis, but also suggested potential drugs to develop more effective cancer treatment strategies for patients carrying the LCT events.

## Conclusions

In this study, we developed a bioinformatic method, ReFuse, to identify and quantify LCTs in bulk and singe cell RNA sequencing data of lung cancer. The LCT events affected genes involved in specific metabolic processes and mitochondrial functions. Function analysis of a tumor specific and frequent LCT involving *FGGY* (*L1-FGGY*) reveal that the AA metabolic pathway was activated by the loss of *FGGY* through the *L1-FGGY* chimeric transcript to promote tumor growth, which was effectively targeted by a combined use of an anti-HIV drug (NVR) and a metabolic inhibitor (ML355). Those findings characterize the role of L1 in metabolic reprogramming of lung cancer and provide rationale for L1-specifc prognosis and potential for a therapeutic strategy for treating lung cancer.

## Data and Method

### L1 and Refseq reference

The repeat gene family annotation for hg38 was downloaded from the RepeatMasker [67] database and repeat families annotated as “LINE/L1” were extracted for further analysis. We then selected the L1 families whose coordinates located within the upstream and downstream 50 kb of coding genes from NCBI Refseq database [68]. The sequences of these L1 families were extracted from the human genome hg38 using bedtools getfasta [69] based on their genome coordinates. makeblastdb of blast 2.2.26 + [70] was used to build the reference for blast mapping. Meanwhile, all the repeat sequences from RepeatMasker were also extracted and genomeGenerate was used to build the reference for STAR mapping to remove false positive chimeric candidates.

Refseq RNA sequences was downloaded from NCBI data base. Repeatmasker 4.0.7 (http://www.repeatmasker.org >) was used to identify and mask the repeat sequences in the RNA sequence using hmmer method. The repeat sequence identified from the RNA sequence was replaced with Ns. Two reference transcriptomes were generated with makeblastdb of blast 2.2.26 + , one with original Refseq sequence and one for masked reference. A bwa reference for the unmasked RNA sequence was also built with bwa index with default parameters [71].

### Refuse strategy

Raw RNA sequences were mapped to the Refseq transcript reference (unmasked) using bwa mem with default parameters. Unmapped reads and soft clipped reads were selected using samtools 1.9 [72] and further aligned to L1 reference sequence using blastn in blast/2.2.26 + with default parameters. Reads with one end partially mapped to L1 family were trimmed and the unmapped end was blast against the Repeat-masked Refseq sequence using blast-short since some fragments might be short in length (Repeat-masked Refseq was used here to avoid false positive reads that mapped to different L1-repeat sequence in two ends). Then the reads that partially aligned to L1 and Refseq was identified. Considering balstn can only handle reads longer than 18 bp, some partially mapped reads might be missed. We then extend the identified reads ReadLength-1 bp along the corresponding L1 and Refseq sequencing from the junction site and constructed a candidate L1-chimeric sequence set. The candidate chimeric sequences are clustered with cd-hit [73] with 100% identity to remove duplicates and then mapped to all repeat sequences using STAR [74] in a gap allowed manner to remove false positive that containing repeat sequence fusions. Finally, all the unmapped reads were blast back to this candidate L1-chimeric sequence set to find more support reads. The final expression value for L1-chimeric transcript was quantified by the counting the supported reads.

### Identification of differentially expressed LCT Events

Raw LCT-sample counts were normalized with the total RNAseq reads in each sample and then log transferred. Then two strategies were used to identify differentially expressed LCT events. 1) limma test [75] was performed on the normalized expression value of LCTs in tumor samples against normal controls. The LCT events with L1 chimeric *p* value <  = 0.05, log2 fold change > 0 were selected as differentially expressed events. 2) For each event, the number of occurrences in cancer and control of LCTs and normal transcripts were counted and Fisher’s exact test was performed to calculate the significance p value. The LCT events with *p* value <  = 0.05 were selected as differentially expressed events. Finally, the union set of differentially expressed events identified in the two strategies were selected as the differentially expressed events.

### Correlation analysis and survival analysis

The “M” methylation value was calculated as described in [76] and the methylation value of each sample was calculated as the median methylation M value of all sites in the sample and the correlation with LCT expression was tested using Pearson correlation test. A sample level copy number was quantified by the sum of absolute Gistic2 copy number value of all the genes in each sample. The correlation of sample level copy number and the LCT expression was tested using Pearson correlation test. A sample level LCT activity was calculated as the sum of LCT supported reads and normalized by the total RNAseq read number of each sample.

All LCT activity related genes were identified by correlating the overall LCT expression level and individual gene expression levels using a Pearson correlation test. LCT correlated genes were identified with *p* value <  = 0.0001 and further divided into positive related (> 0) and negative related (< 0) based on the estimated correlation value by Pearson correlation test between sample-level LCT and gene log_2_ FPKM expression. The Cox model was used for survival analysis.

### Immune markers and GSEA analysis

In-house markers for immune cells populations were identified based on 7 individual PBMC single cell and 5 bulk RNAseq data sets of sorted immune cell. For each cell type, their expression profile was compared against other cell types one by one with Wilcoxon rank sum test. Genes with *p* value < 0.01 and mean log_2_ fold change larger than 0 in all comparisons was selected as the specific markers of the cell type. Each cell type was calculated in the same way in each sample and markers identified more than 3 times was selected as the meta-marker for the cell type. A “gmt” file for GSEA analysis was then constructed using the identified meta marker list [77]. GSEA analysis was done by “fgsea” R package with fold change for each gene calculated using limma [75].

### Single cell clustering, trajectory and differential analysis

Expression profile of raw gene counts on cell level of each sample was downloaded from Array Express. Genes expressed in less than 3 cells and Cells expressing more than 8000 genes were filtered. Cells whose mitochondrial RNA content takes larger than 15% of the total RNA were filtered. Filtered cells are further clustered using Seurat 3.1.5 [78] with resolution 0.8 and first 10 PCA dimensions, and cell clusters were annotated based on the markers in the original paper and another single cell papers on lung cancer [79]. Differentially expressed genes between cell types and L1versus non-L1 cells were identified using Wilcoxon rank sum test with p value less than 0.01 and log (fold change) greater than 0.25.

### Apply refuse on single cell RNAseq data

Raw sequence data of the single cell study were downloaded from Array Express. The file containing RNA sequence reads (R2 for 10 × v2 and R1 for 10 × v3) of each sample was submitted to run on Refuse to identify LCT events as single end bulk RNAseq data. The barcodes of LCT supporting reads were extracted in the barcode sequencing file and clustered using CD-hit [73] using end-to-end mode with identity score larger than 0.85 to allow for sequencing errors. Barcodes clustered together were annotated as the same cell and the barcode found in expression profiles was selected as the representative barcode of the cell cluster.

### LCT affected candidate genes and drug repurposing

Genes identified to be positively correlated with overall survival related LCT activity (*p* <  = 0.0001 and estimate > 0) and differentially expressed by cells with L1 versus without L1 in > 3 cell types/patients (myeloid, T cell and tumor cell in 5 patients, totally 15 comparisons) were selected as candidate genes affected by LCT. Those genes were submitted to the CMAP database [80] for drug repurposing for potential treatment on patients with high LCT activity.

The log_2_ RPKM gene expression in all the patients of each candidate gene was z-normalized, and an aggregate score was calculated by summing up the z score of all the 47 candidate genes for each sample. The patients were divided into 4 groups with the threshold of aggregate gene score and survival related LCT expression. By iterating the two thresholds, a high-risk group was identified having 50% fatality rate within 3 years, which could be highly related to LCT activity.

### Potential treatment for LCT related patients

Raw sequence data of cancer cell line treated with Metformin and simvastatin were generated from NCBI GEO data base with accession number GSE146982, GSE141052 and GSE149566 [81, 82]. LCT analysis was performed with ReFuse and the overall LCT levels was summarized by adding up all the junction reads supporting LCT events and normalized with the read depth in each sample. For simvastatin data sets, few LCT were found as it is not from a cancer cell line, so we quantified the L1 activity with L1 reference sequence. First, the raw sequences were mapped to Refseq using bwa mem 0.7.15 [71] and the unmapped reads were then mapped to L1 reference using STAR 2.7.0f [74]. The overall L1 activity was quantified by counting all the reads mapped to L1 reference and normalized by the read depth in each sample.

### Patient information

All the LUSC patients were obtained from Cancer Biobank of Tianjin Medical University Cancer Institute and Hospital (TJMUCH, Table S20-S21) which were treated with partial lung resection surgery at the Department of Lung Cancer of TJMUCH. No prior treatments, including chemotherapy or radiotherapy, were conducted before lung resection surgery was performed. This project was approved by the Ethics Committee of Tianjin Medical University (Approved No.: Ek2020111) and written informed consents were obtained from the patients. All experiments were performed in accordance with the principles of the Declaration of Helsinki.

### Cell lines

NCI-H520 and SK-MES-1 were purchased from Cellcook Co., Ltd. with cell authentication via STR multi-amplification method. KLN205 was obtained from Chinese Academy of Medical Sciences tumor cell libraries. NCI-H520 was cultured in RPMI1640 (Gibco BRL). SK-MES-1 was cultured in MEM. KLN205 was cultured in H-DMEM. All medium contained 10% FBS and 1% penicillin/streptomycin. For 12/15-LOX inhibitor treatment experiments, ML355 and PD146176 was diluted in medium, followed by replacing the cell medium 5 h after cells seeded respectively. Cell lines were routinely evaluated for Mycoplasma contamination. All experiments were completed less than 2 months after establishing stable cell lines or thawing early-passage cells.

### Mouse models

The DBA2/2NCrl mice are an inbred line and were obtained from SPF biotechnology Co. Ltd. (Beijing). The weights and tumor sizes of each mouse were monitored every 2 days. Each experimental group contained 5 mice. The tumor volume (V) of the xenograft was calculated by the formula: V = π × L × W × H/6 (L: length, W: width, H: height). For drug treatment studies, animals were subjected to treatment with either NVR or ML355 every day, or subjected with the two inhibitors simultaneously. All animal protocols were approved by the Ethics Committee for Animal Experiments of TJMUCH (Approved No.: NSFC-AE-2020101), and was performed in accordance with the Guide for the Care and Use of Laboratory Animals.

### Lentivirus construction

The construction of human *L1-FGGY* insertion lentivirus was performed as previously described [19]. For mouse *L1-FGGY* insertion lentivirus construction, the recombinant lentivirus with *L1-FGGY* sequence was generated by co-transfection in the packaging KLN205 cells. For *GPR31*/*USP24* knockdown lentivirus construction, the recombinant lentivirus with *GPR31*/*USP24* shRNA sequence (constructed by Hanbio Co., Ltd.) was generated by co-transfection in H520^OV−*L1−FGGY*^ cells as previously described [19].

### RNA extraction, Reverse Transcription-Polymerase Chain Reaction (RT-PCR), and quantitative Real-time PCR (qPCR) analysis

RNA was extracted with TRIzol™ reagent (Life Technologies). Reverse transcription was performed with PrimeScript™ RT Master Mix (Takara) according to the manufacturer’s instructions. qPCR was performed using TB Green™ Premix Ex Taq™ (Takara) and ABI PRISM 7500 real-time PCR System (Applied Biosystems). The primers used are shown in Table S23. The relative mRNA levels were calculated as previously described [19].

### RNA library preparation, sequencing and enrichment analysis

Library preparation and sequencing steps were performed as previously described [19]. Briefly, the libraries were sequenced on Illumina® (NEB) following manufacturer’s recommendations. The RNAseq data has been uploaded to GEO database (accession number: GSE181042 and GSE181043). Raw sequencing data was aligned to hg38 reference using STAR 2.5.3a [74] and HTseq 0.11.2 [83] was used to quantify the gene-sample expression profiles. Differentially expressed genes (DEG) were identified with limma voom [84] with FDR adjusted *p* value < 0.01. KEGG and GO function enrichment analysis of the interested gene sets were performed using clusterProfiler package [85].

### Quantitative proteomics

The quantitative proteomic studies were performed by Jingjie PTM BioLab Co. Ltd (Hangzhou) as previously described [86]. Briefly, the protein extracted was digested and then the resulting peptides were desalted, reconstituted, tandem mass tag labeled, and analyzed by Liquid chromatography-tandem mass spectrometry (LC–MS/MS). Tandem mass spectra were searched against human Uniprot database (http://www.ebi.ac.uk/uniprot/) concatenated with reverse decoy database.

### Metabolite analysis targeting arachidonic acid (AA) signaling

AA metabolite detection was performed by Shanghai Applied Protein Technology Co., Ltd. Cells were homogenized on ice in a mixture of chloroform, methanol and water. The samples were then centrifuged and the supernatant was transferred to an LC sampling vial. The deposit was rehomogenized with methanol and supernatant was added to the same vial. After reconstituted with mobile phase, the extract as well as reference standards were analyzed with ACQUITY ultra performance liquid chromatography coupled with mass spectrometer (Waters). UPLC-MS raw data obtained with negative mode were analyzed using TargetLynx applications manager to obtain calibration equations and the quantitative concentration of each AA metabolite in the samples.

### Gene expression (RNA) profiling: NanoString methodology

Gene expression analysis was conducted on the NanoString nCounter gene expression platform (NanoString Technologies) as previously described [87]. Briefly, RNA was mixed in a 3′-biotinylated capture probe and 5′-reporter probe tagged with a fluorescent barcode. Probes and target transcripts were hybridized overnight. Hybridized samples were run on the NanoString nCounter preparation station by using a high-sensitivity protocol. The cartridge samples were scanned at maximum resolution by using the nCounter digital analyzer. GEP scores were calculated as a weighted sum of normalized expression values for the genes.

### Cell proliferation assay

The cell proliferation was detected by Cell Counting Kit 8 (CCK8) proliferation assay as previously described [19]. Briefly, cells were trypsinized and incubated with CCK8 for 4 h. Then the absorbance reading at 450 nm was taken by a microplate reader (Synergy HT).

### Wound healing assay

The wound healing assay was performed as previously described [19]. Briefly, when the seeded cells reached 80 ~ 90% confluency, we made a straight line in the cell monolayer. At 0 and 48 h, images were obtained, and the distance of the wound was measured.

### Transwell invasion assay

The transwell invasion assay was performed as previously described [19]. Briefly, we seeded cells in Matrigel and serum-free RPMI-1640. Medium supplemented with 10% FBS was placed in the lower chamber of the Transwell. After 48 h’ incubation, we fixed the cells on the membrane’s lower surface and subjected them to staining with 1% toluidine blue. After staining photographs were taken under a microscope, the number of invading cells was recorded.

### Enzyme-linked immunosorbent assay (ELISA)

The levels of 5S-HETE, 12S-HETE, 15S-HETE, PGD2, PGE2, PGF2, Wnt3a and Wnt5a either in cell culture supernatants or from tissue samples were measured using commercially available ELISA kits (Abcam and Bioswamp) according to the manufacturer’s instructions.

### Western blot

Proteins were electrophoresed by SDS/PAGE and blots were incubated overnight with primary antibody. The following antibodies were used: anti-GPR31 (Abcam, ab75579), anti-ATPase Na^+^/K^+^ β2 (Bioss, bs-1152R), anti-Wnt3a (Cell signaling Technology (CST), #2391), anti-Wnt5a (CST, #2530), anti-pGSK-3β (phospho Ser9, CST, #5558), anti-JNK (phospho Thr183/Tyr185, CST, #4671), anti-β-catenin (CST, #8480), anti-HA tag (CST, #5017), anti-flag tag (CST, #14,793), anti-USP24 (Proteintech, 13,126–1-AP), anti-FGGY (Abnova, ABN-H00055277), anti-β-Actin (CST, #4967), and anti-GAPDH (Abcam, ab181602). After incubated with HRP-conjugated α-rabbit or α-mouse secondary antibodies for 1 h, protein bands were detected with chemiluminescence substrate (Perkin Elmer) using the ChemiDoc Imaging System (Bio-Rad).

### Immunoprecipitation

Cell lysates were harvested using lysis buffer, rotated at 4 °C and as previously described [88]. Lysates were clarified by spinning. Protein concentrations were measured using BCA standard curves (Pierce). Flag antibody (for binding to flag-GPR31, Thermo Fisher Scientific) was added to protein lysate and rotated overnight. IP was carried out using the Invitrogen Dynabeads Protein G Immunoprecipitation Kit as directed. Lysates were next subjected to SDS-PAGE and immunoblot analysis. Each immunoprecipitation experiment was performed a minimum of twice.

### Immunohistochemistry

All procedures were performed as described above [89]. The antibodies are as follows: anti-FGGY (Abcam), anti-12-LOX (Abcam), anti-15-LOX (Abcam), anti-GPR31 (Abcam), and a biotinylated secondary goat anti-mouse IgG antibody (Santa Cruz), labeled with HRP using a DAB staining kit (Maixin Biotechnology) according to the manufacturer’s instructions. For negative controls, IgG1 was used. Positively stained cells were counted in 5 fields at 200 × magnification.

### Flow cytometry

Cells were incubated with different antibodies for 30 min at 4 °C as indicated. The following antibodies were used: PerCP anti-mouse CD45, APC anti-mouse CD3, FITC anti-mouse CD4, PE anti-mouse CD8, PE anti-mouse CD11c, and FITC anti-mouse CD86. We selected isotype-matched immunoglobulin G1 antibodies (BD Biosciences) to serve as a negative control. The cells were analyzed on a BD FACS CantoTM II flow cytometer and FlowJo software (BD Biosciences).

### Multispectral immunofluorescence (IF) staining

We performed multispectral IF staining as previously described [90]. In brief, the slides were deparaffinized and rehydrated. After antigen retrieval and blocking, the primary antibody was applied and incubated overnight. Opal polymer HRP was used as the secondary antibody. The slides were washed, and tyramide signal amplification (TSA) dye (PerkinElmer) was applied. The slides were then exposed to microwaves to remove the primary and secondary antibodies, washed, and blocked again. Afterward, other primary antibodies, as well as DAPI were applied successively. Finally, slides were placed on a coverslip. Five fields at 200 × magnification was imaged and recorded, and StrataQuest Image Analysis software was used to generate a spectral library for unmixing.

### Transmission electron microscopy (TEM)

Cells were fixed with 2.5% glutaraldehyde, postfixed with 0.5% osmium tetroxide and contrasted using tannic acid and uranylacetate. Specimens were dehydrated in a graded ethanol series and embedded in Polybed. Ultrathin sections were analyzed in a HT7800 transmission electron microscope.

### Measurements of oxygen consumption and extracellular acidification

The rates of oxygen consumption rate (OCR) and extracellular acidification rate (ECAR) in various cell lines were measured with a Seahorse Bioscience XF-24 extracellular flux analyzer, as detailed previously [91, 92]. Cells were seeded at a density of 1 × 10^4^ cells per well on Seahorse XF-24 polystyrene tissue culture plates. Inhibitors were used at the following concentrations: Oligomycin (1.5 μM), Carbonyl cyanide 4-trifluoromethoxy-phenylhydrazone (FCCP) (0.8 μM), Antimycin A (1.5 μM) and Rotenone (3 μM).

### Assessment of mitochondrial membrane potential (MMP)

JC-1 was used to measure the MMP according to the manufacturer’s instructions (Bioss). Cells in 6-well plates were incubated with JC-1 staining solution at 37 °C for 30 min and then washed with JC-1 buffer. Fluorescent signals were obtained using flow cytometry.

### Measurement of intracellular ATP levels

ATP levels were measured using the Enhanced ATP Assay Kit (Beyotime Biotechnology) according to the manufacturer’s instructions. The substrate was gently mixed with reaction reagent at room temperature. The luminescence was then measured using a Beckman Coulter.

### Statistical analysis

Data were statistically analyzed with SPSS 20.0 and GraphPad Prism 5.0 software following the manufacturers’ instructions. Measurement data were expressed as means ± standard deviations. We analyzed correlations between 2 datasets using Spearman’s correlation coefficient. One- and two-way analysis of variance with subsequent Bonferroni post-hoc tests was used for comparisons between 2 groups. Cumulative survival was determined via the Kaplan–Meier method. All data were normally distributed. *P* < 0.05 was taken to indicate a statistically significant result.

## Supplementary Information

**Additional file 1.** **Figure S1.** Validation of LCT activity in TJMUCH cohort.(A) Number of LCT events overlapped between TJMUCH cohort and TCGA LUSC cohort.(B) Comparison of overall methylation level between LUSC and LUAD patients fromTCGA. (C) Distribution of LCT event number in each sample. (D) Expression (readper mission) between tumor and control samples. (E) Frequency of the top 20 LCTevents (named by their target genes). (F) Enriched GO terms of the recurrentLCT affected genes (more than 2 samples). (G) Differentially expressed LCTsbetween tumor and normal samples. Left panel showing the number of samplesdetected with each LCT event and the right panel showing the expression ofcorresponding LCT event across samples (red represents tumor samples while bluerepresents normal samples). **Figure S2. **Association of LCT with Metabolic, Immuneprocess, Genomic Instability and Clinical States. (A) Enriched GO terms ofgenes positively or negatively co-expressed with overall LCT expression in eachsample. (B) The GSEA enrichment plot for meta-markers of each immune cell type.Genes are ranked by the fold change of expression in LCT-high samples (left)versus LCT-low samples (right) (C) Correlation of methylation level and CNVlevel with overall LCT activity in each sample. (D) Comparison of LCTexpression in different cancer stages and TMB quartiles. (E) Differentiallyexpressed LCT events (named by targeted gene), which are highly expressed intumor than control. Left panel shows the number of samples containing the LCT eventwhile right panel shows the expression of LCT events among all the samples,grouped by tumor (red) and normal control (blue). (F) Survival curve ofpatients grouped as L1-high and L1-low by the total expression of survivalrelated LCTs. **Figure S3. **Validation of the association of overall LCTactivity and Immune function in TJMUCH cohort. (A) Enriched GO terms of thegenes positively co-expressed with overall LCT activity. (B) Enriched GO termsof the genes negatively co-expressed with LCT activity. (C) GSEA analysis ofthe immune markers enrichment in LCT-high versus LCT-low samples. **Figure S4. **LCT activity in tumor cells. (A) Frequencyof overlapped LCT between bulk and single cell data sets. Left panel shows thefrequency of each LCT event in each cancer type from TCGA bulk sequencing.Right panel shows the occurrence of each LCT event in each sample from singlecell data set. (B) Umap of cells colored by individual. (C) Markers for annotationof each cell type. (D) the proportion of each cell type in each sample. **Figure S5. **LCT related DEGs between cellpopulation. (A) Umap and trajectory of tumor cells fromPatient1, Patient2 and Patient5. Cells detected with LCT events were coloredred. (B) DEGs between LCT enriched tumor subtype versus other tumor cells. (C)DEGs between LCT positive tumor cells versus LCT negative tumor cells in LCTenriched tumor subtype. **FigureS6. **The mitochondrialmorphology and functions. (A) The TEM results of H520^OV-CTRL^, H520^OV-*L1-FGGY*^, and BEAS-2B. (B) The OCR and ECAR results ofH520^OV-CTRL^and H520^OV-*L1-FGGY*^. (C) The MMP results of H520^OV-CTRL^and H520^OV-*L1-FGGY*^.(D) The ATP productionof H520^OV-CTRL^and H520^OV-*L1-FGGY*^. **Figure S7. **The oncogenic roles of *L1-FGGY*/12-LOX/GPR31in SK-MES-1 cells. (A) The relative RNA expression of 12-LOX and 15-LOXdetected in SK-MES-1^OV-CTRL^ and SK-MES-1^OV-*L1-FGGY*^, as well as in SK-MES-1^OV-*L1-FGGY*^ treated with either ML355 or PD146176. (B) The secretionvalue of 12S-HETE and 15S-HETE detected in SK-MES-1^OV-CTRL^, SK-MES-1^OV-*L1-FGGY*^, and SK-MES-1^OV-*L1-FGGY*^ treated with either ML355or PD146176. (C) The proliferation of SK-MES-1^OV-CTRL^, SK-MES-1^OV-*L1-FGGY*^, SK-MES-1^OV-*L1-FGGY*+sh-*GPR31*^, and SK-MES-1^OV-*L1-FGGY*^ treated with either ML355 or PD146176 was detectedusing CCK8 method. (D) The statistical results of migration rates of SK-MES-1^OV-CTRL^,SK-MES-1^OV-*L1-FGGY*^,SK-MES-1^OV-*L1-FGGY*+sh-*GPR31*^, and SK-MES-1^OV-*L1-FGGY*^ treated with either ML355or PD146176 in wound healing assays. (E) The statistical results of invasionnumber of SK-MES-1^OV-CTRL^, SK-MES-1^OV-*L1-FGGY*^, SK-MES-1^OV-*L1-FGGY*+sh-*GPR31*^, and SK-MES-1^OV-*L1-FGGY*^ treated with either ML355or PD146176 in trans-well invasion assays. The data are shown as mean ± SD withplots. * and ** indicate *p*<0.05 and *p*<0.01, respectively between thegroups as indicated. **Figure S8.** The results of nCounter® PanCancer IO-360™Panel detected in *L1-FGGY*^+^tissues and *L1-FGGY*^-^tissues (*n*=12). (A) The pathway scores of different signaling pathwayswere compared between *L1-FGGY*^+^tissues and *L1-FGGY*^-^tissues. (B) The cell type scores of immunocytes were compared between *L1-FGGY*^+^ tissues and *L1-FGGY*^-^ tissues. **Figure S9. **The model of *L1-FGGY*activated 12-LOX/GPR31/Wnt signaling through competitive combination-inducedUSP24-mediated deubiquitination.** Figure S10. **NVR and ML355 triggered metabolism reprogramming andaltered the immune microenvironment *invivo*. (A) The qPCR results of genes involved inglucose and lipid metabolism were shown as relative expression values in KLN205^OV-CTRL^mouse group, KLN205^OV-*L1-FGGY*^mouse group, NVR-treated KLN205^OV-*L1-FGGY*^mice, ML355-treated KLN205^OV-*L1-FGGY*^mice and NVR+ML355-treated KLN205^OV-*L1-FGGY*^mouse group. (B) The proportions of various immunocytes in different mousegroups using flow cytometry.** Figure S11. **Potential effects of drug on LCT activity. (A) Distribution of LCT expression in samples treatedwith Metaformin and control samples in two public data sets. (GSE141052 andGSE146982) (B) Distribution of L1 expression in samples treated withSimvastatin and control samples in two pancreatic cancer cell line (PANC-1 andMiaPaCa-2 (GSE149566)). **Table S1. **Basic statistics of LCT events in all 3cohorts. **Table S2. **Overlapped LCT genes between TCGA LUSCand LUAD.** Table S3. **Overlapped LCTgenes between TCGA and TJMUCH LUSC samples.** Table S4. **Overlapped GO BPterms enriched with recurrent LCT genes between TCGA LUSC and LUAD.** Table S5. **Enriched GO BPterms with recurrent LCT genes in TJMUCH cohort.** Table S6. **GO BP termsenriched with genes positively co-expressed with overall LCT level in TCGA LUSCand LUAD samples.** Table S7. **GO BP termsenriched with genes negatively co-expressed with overall LCT level in TCGA LUSCand LUAD samples.** Table S8. **GO BP termsenriched with genes positively co-expressed with overall LCT level in TJMUCHLUSC samples.** Table S9. **GO BP termsenriched with genes negatively co-expressed with overall LCT level in TJMUCHLUSC samples.** Table S10. **Differentiallyexpressed LCTs between tumor and normal samples in TCGA LUSC.** Table S11. **Differentiallyexpressed LCTs between tumor and normal samples in TJMUCH LUSC.** Table S12. **Differentiallyexpressed LCTs between tumor and normal samples in TCGA LUAD.** Table S13. **Survival relatedLCTs in TCGA LUSC.**Table S14. **Survival relatedLCTs in TCGA LUAD. **Table S15. **Recurrent LCTsbetween bulk RNAseq from TCGA and 19 SC RNAseq samples. **TableS16.** Differentially expressed genes between LCT enriched tumor cell cluster andother tumor cell clusters in each patient. **TableS17.**GO BP enrichment of differentially expressed genes between LCT enrichedtumor cell cluster and other tumor cell clusters in each patient. **Table S18. **Differentiallyexpressed genes between tumor cells with LCT versus tumor cells without LCT inLCT enriched tumor cell cluster in each patient. **TableS19.** GO BP enrichment of differentially expressed genes between tumor cells withLCT versus tumor cells without LCT in LCT enriched tumor cell cluster in eachpatient. **Table S20.** The basic clinical pathologicalinformation of all TJMUCH patients. **Table S21.**Distributions of clinical-pathological parameters of patients with *L1-FGGY*^+^and *L1-FGGY*^-^ of TJMUCH.**TableS22.** List of 47 LCT related genes selected for grouping of patients.**TableS23.**The sequences of primers in RT-qPCR analysis.

## Data Availability

RNA sequencing data was deposited on GEO database under accession number: GSE181042 and GSE181043.

## Abbreviations

LINE-1, L1: Long Interspersed Nuclear Element-1
LCT: L1-gene chimeric transcript
LRT: L1 retrotransposition
L1-ASP: L1 antisense promoter
LTR: Long-terminal repeat
TE: Transposable elements
TIME: Tumor stage and tumor immune microenvironment
AA: Arachidonic acid
NSCLC: Non-small cell lung cancer
LUAD: Lung adenocarcinoma
LUSC: Lung squamous cell carcinoma
WES: Whole exome sequencing
ReFuse: Retrotransposon-gene fusion estimation program
TJMUCH: Tianjin Medical University Cancer Institute and Hospital
DEG: Differential expressed gene
LOX: Lipoxygenase
COX: Cyclooxygenase
CYP450: Cytochrome P450
ELISA: Enzyme-linked immunosorbent assay
WCR: Wound closure rate
TILs: Tumor infiltrating lymphocyte
DC: Dendritic cells
GPCR: G protein-coupled receptor
TEM: Transmission electron microscopy
RT-PCR: Reverse Transcription-Polymerase Chain Reaction
qPCR: Quantitative Real-time PCR
CCK8: Cell Counting Kit 8
IF: Immunofluorescence
TSA: Tyramide signal amplification
OCR: Oxygen consumption rate
ECAR: Extracellular acidification rate
FCCP: 4-Trifluoromethoxy-phenylhydrazone
MMP: Membrane potential
