# Supplementary material for: LINE-1 promotes tumorigenicity and exacerbates tumor progression via stimulating metabolism reprogramming in non-small cell lung cancer
Source: Mol Cancer. 2022 Jul 16;21:147. doi: 10.1186/s12943-022-01618-5 (PMC9288060; doi:10.1186/s12943-022-01618-5)
Supplement: Supplementary file 1 — Additional file 1. Figure S1. Validation of LCT activity in TJMUCH cohort.(A) Number of LCT events overlapped between TJMUCH cohort and TCGA LUSC cohort.(B) Comparison of overall methylation level between LUSC and LUAD patients fromTCGA. (C) Distribution of LCT event number in each sample. (D) Expression (readper mission) between tumor and control samples. (E) Frequency of the top 20 LCTevents (named by their target genes). (F) Enriched GO terms of the recurrentLCT affected genes (more than 2 samples). (G) Differentially expressed LCTsbetween tumor and normal samples. Left panel showing the number of samplesdetected with each LCT event and the right panel showing the expression ofcorresponding LCT event across samples (red represents tumor samples while bluerepresents normal samples). Figure S2. Association of LCT with Metabolic, Immuneprocess, Genomic Instability and Clinical States. (A) Enriched GO terms ofgenes positively or negatively co-expressed with overall LCT expression in eachsample. (B) The GSEA enrichment plot for meta-markers of each immune cell type.Genes are ranked by the fold change of expression in LCT-high samples (left)versus LCT-low samples (right) (C) Correlation of methylation level and CNVlevel with overall LCT activity in each sample. (D) Comparison of LCTexpression in different cancer stages and TMB quartiles. (E) Differentiallyexpressed LCT events (named by targeted gene), which are highly expressed intumor than control. Left panel shows the number of samples containing the LCT eventwhile right panel shows the expression of LCT events among all the samples,grouped by tumor (red) and normal control (blue). (F) Survival curve ofpatients grouped as L1-high and L1-low by the total expression of survivalrelated LCTs. Figure S3. Validation of the association of overall LCTactivity and Immune function in TJMUCH cohort. (A) Enriched GO terms of thegenes positively co-expressed with overall LCT activity. (B) Enriched GO termsof the genes negatively [file 12943_2022_1618_MOESM1_ESM.docx]

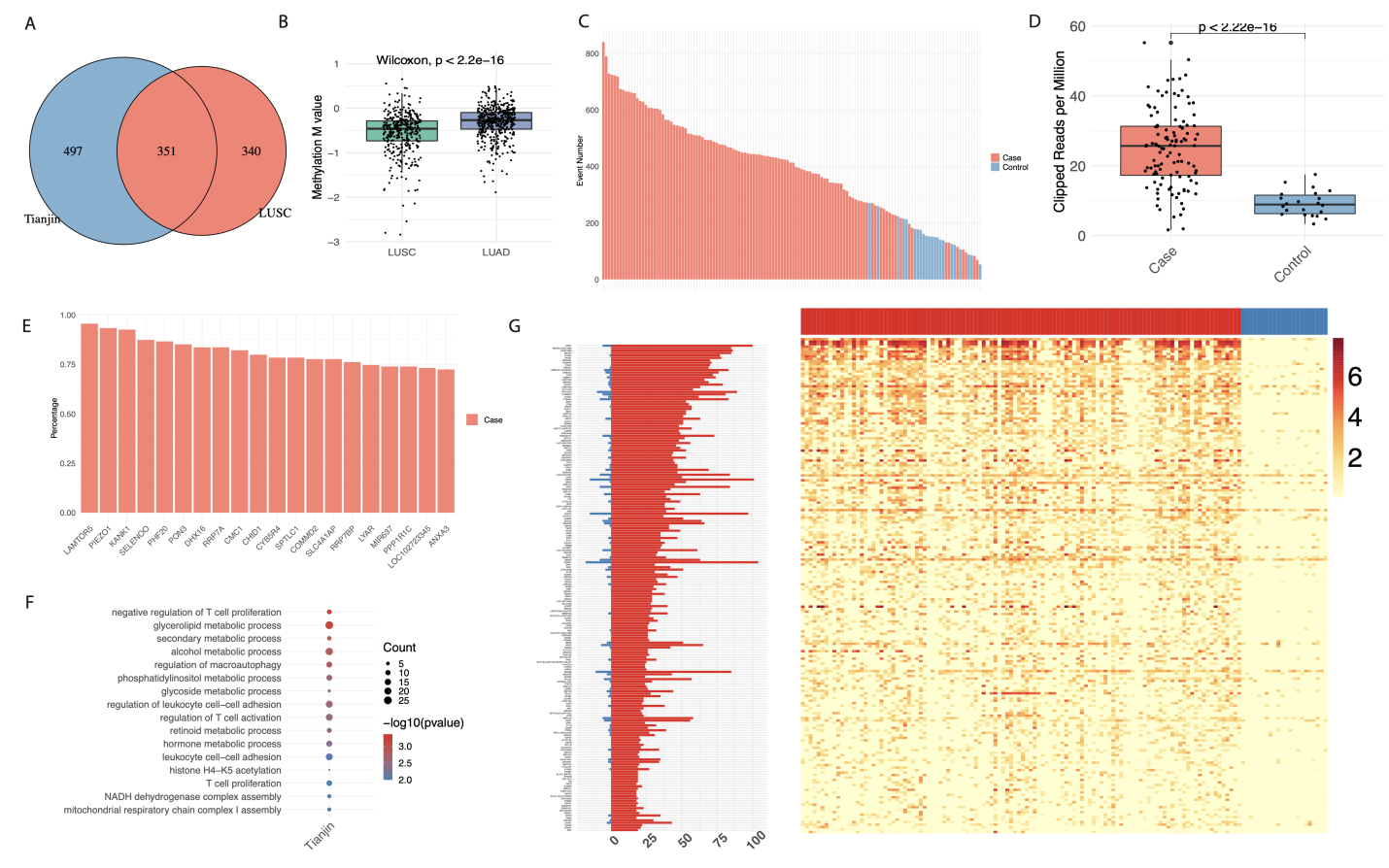


**Figure S1. Validation of LCT activity in TJMUCH cohort.** (A) Number of LCT events overlapped between TJMUCH cohort and TCGA LUSC cohort. (B) Comparison of overall methylation level between LUSC and LUAD patients from TCGA. (C) Distribution of LCT event number in each sample. (D) Expression (read per mission) between tumor and control samples. (E) Frequency of the top 20 LCT events (named by their target genes). (F) Enriched GO terms of the recurrent LCT affected genes (more than 2 samples). (G) Differentially expressed LCTs between tumor and normal samples. Left panel showing the number of samples detected with each LCT event and the right panel showing the expression of corresponding LCT event across samples (red represents tumor samples while blue represents normal samples).


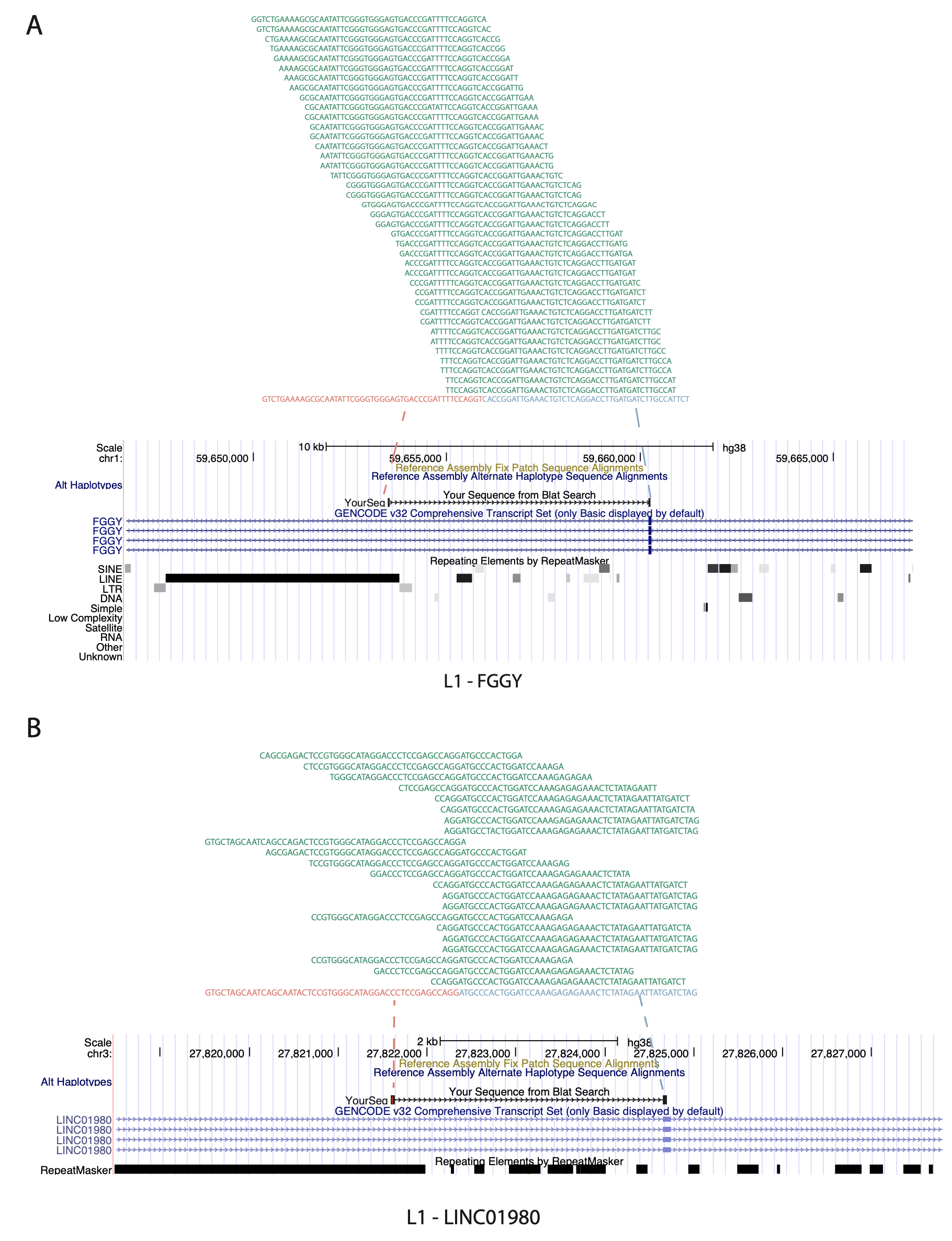


**Figure S2. Demonstration of *L1-FGGY* (A) and *L1-LINC01980*** (B) LCT events. Top panel shows the sequencing reads supporting the event. Middle panel shows the sequence of LCT event (red represents repeat sequence while blue represents coding sequence). Lower panel shows the position of the LCT events and its annotation on UCSC genome browser.


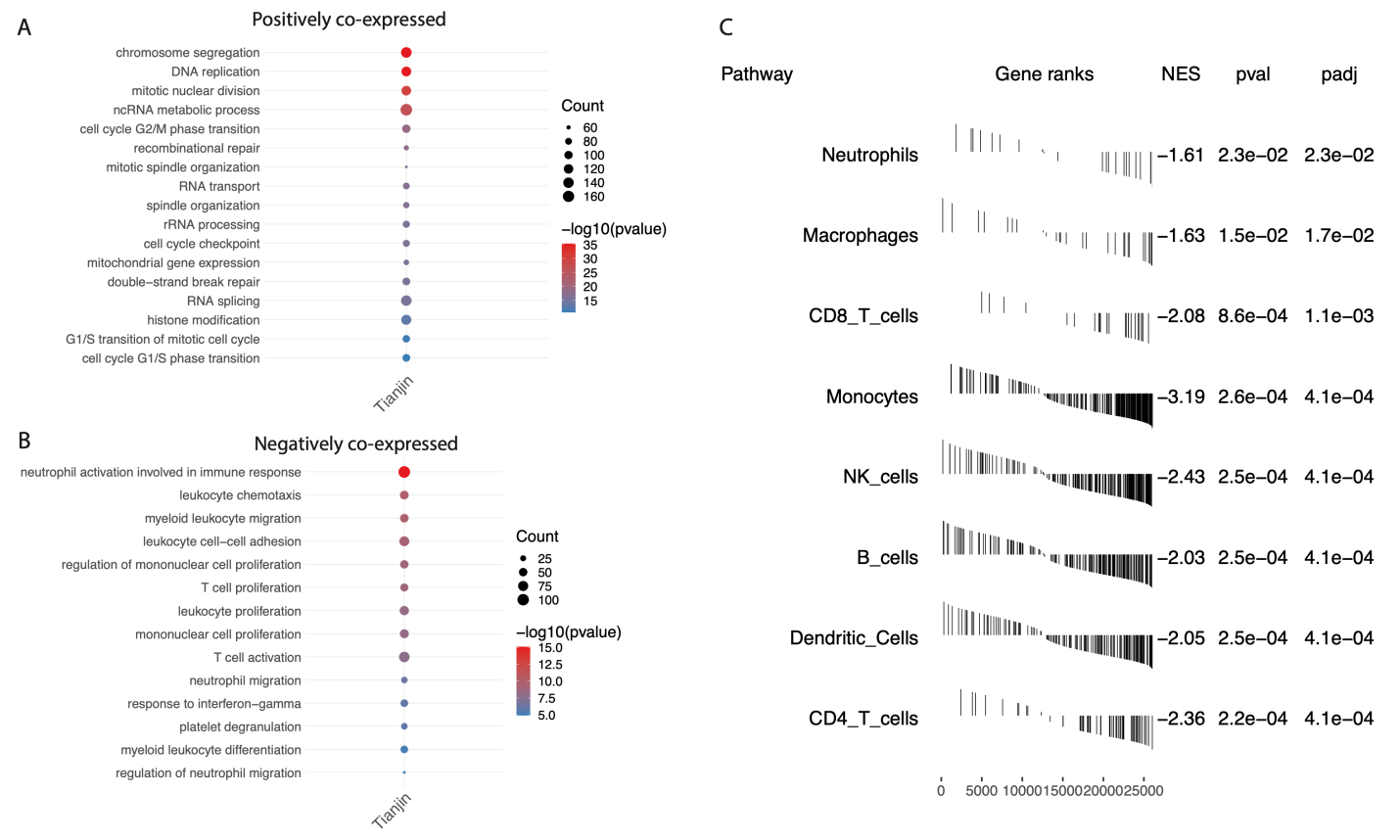


**Figure S3. Validation of the association of overall LCT activity and Immune function in TJMUCH cohort.** (A) Enriched GO terms of the genes positively co-expressed with overall LCT activity. (B) Enriched GO terms of the genes negatively co-expressed with LCT activity. (C) GSEA analysis of the immune markers enrichment in LCT-high versus LCT-low samples.


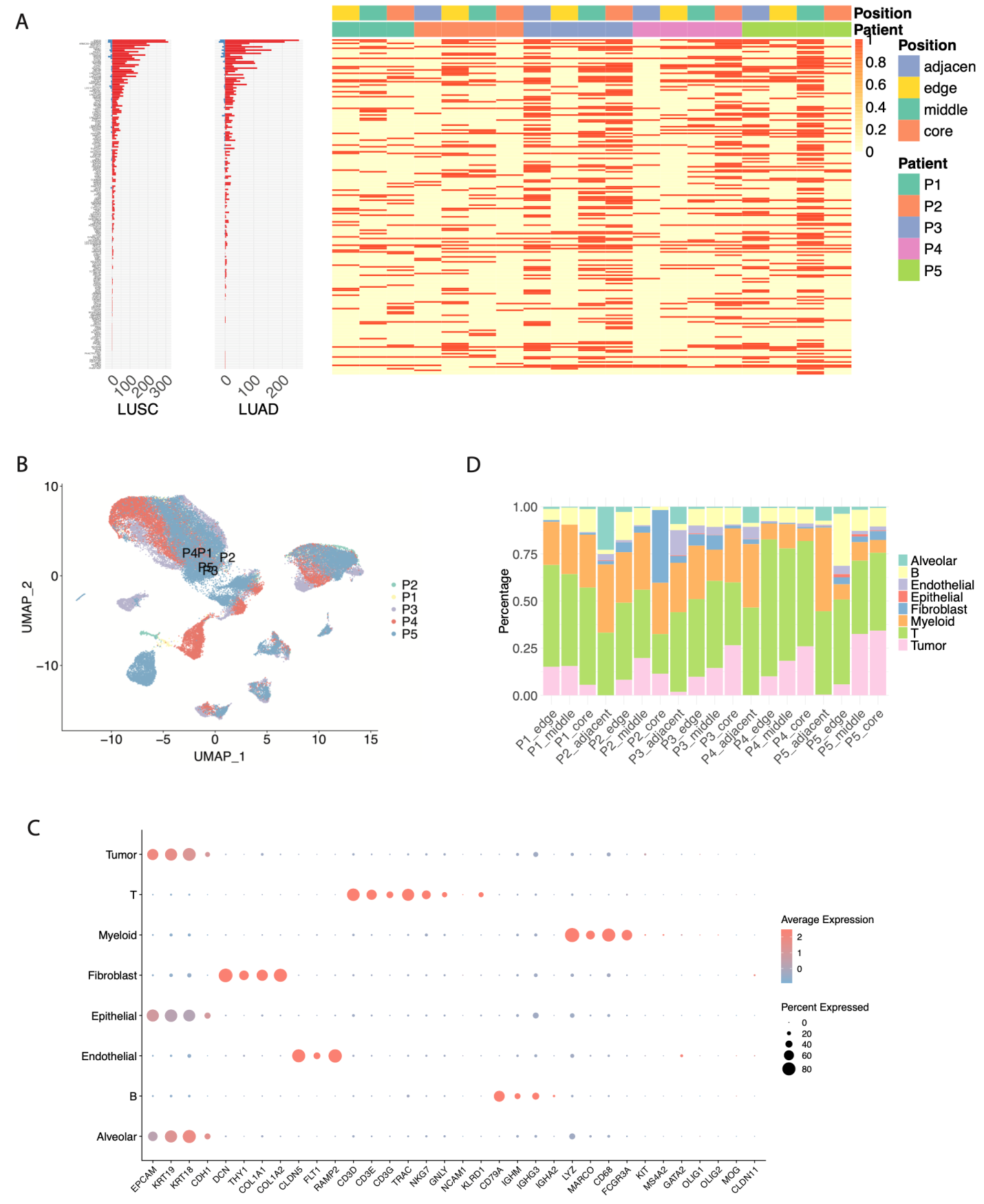


**Figure S4. LCT activity in tumor cells.** (A) Frequency of overlapped LCT between bulk and single cell data sets. Left panel shows the frequency of each LCT event in each cancer type from TCGA bulk sequencing. Right panel shows the occurrence of each LCT event in each sample from single cell data set. (B) Umap of cells colored by individual. (C) Markers for annotation of each cell type. (D) the proportion of each cell type in each sample.


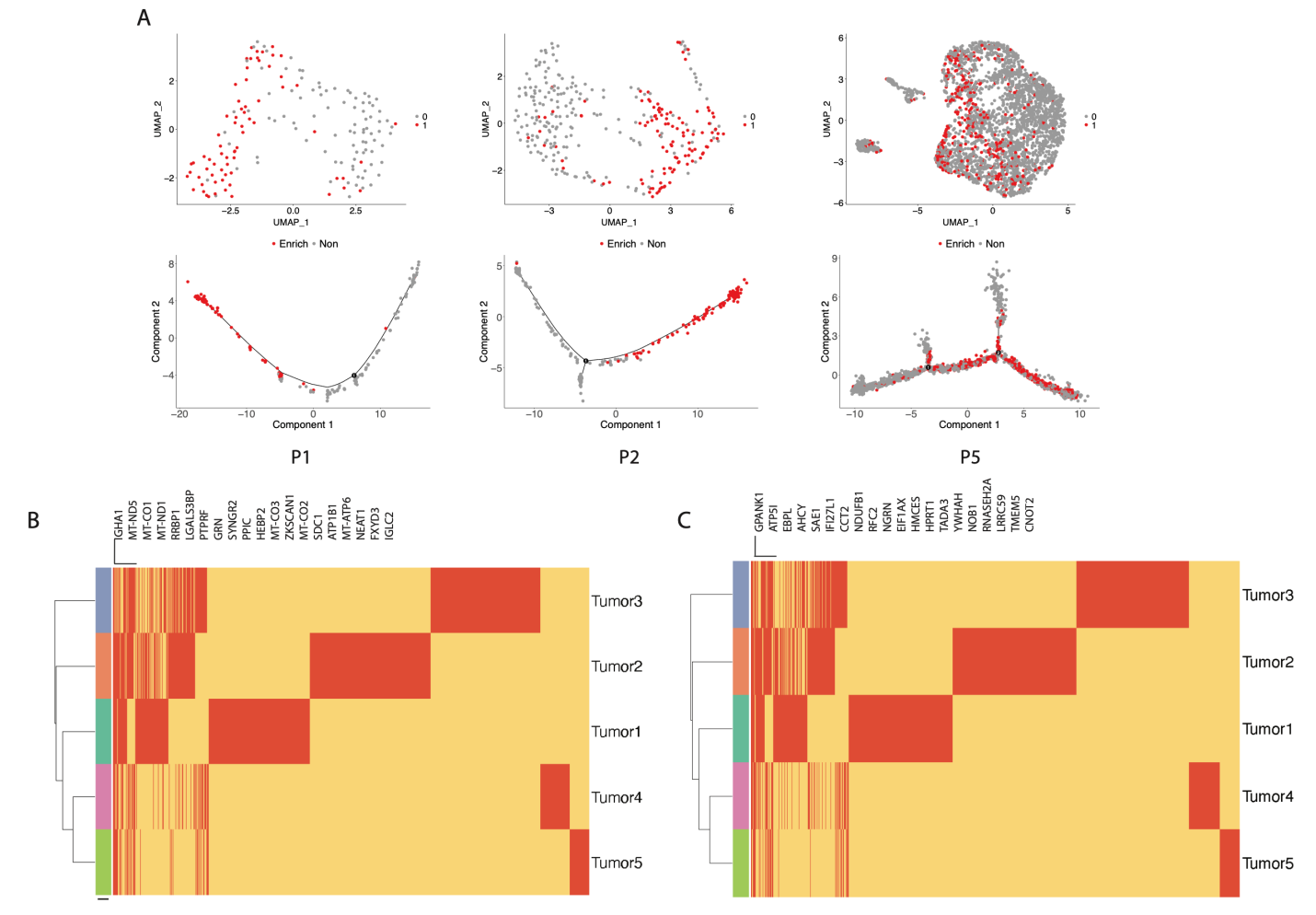


**Figure S5. LCT related DEGs between cell population.** (A) Umap and trajectory of tumor cells from Patient1, Patient2 and Patient5. Cells detected with LCT events were colored red. (B) DEGs between LCT enriched tumor subtype versus other tumor cells. (C) DEGs between LCT positive tumor cells versus LCT negative tumor cells in LCT enriched tumor subtype.


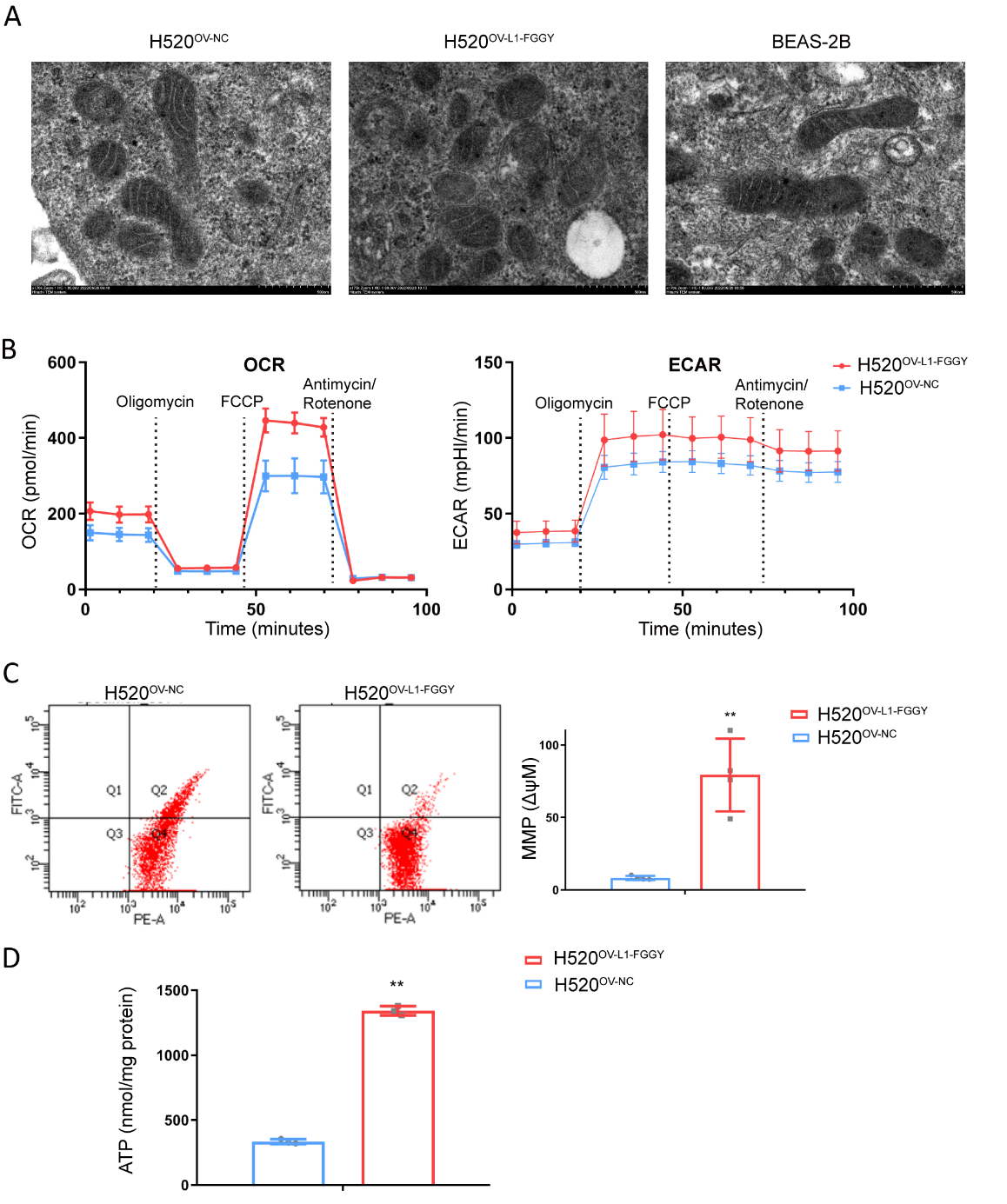


**Figure S6. The** **mitochondrial morphology and functions.** (A) The TEM results of H520^OV-CTRL^, H520^OV-^*^L1-FGGY^*, and BEAS-2B. (B) The OCR and ECAR results of H520^OV-CTRL^ and H520^OV-^*^L1-FGGY^*. (C) The MMP results of H520^OV-CTRL^ and H520^OV-^*^L1-FGGY^*. (D) The ATP production of H520^OV-CTRL^ and H520^OV-^*^L1-FGGY^*.


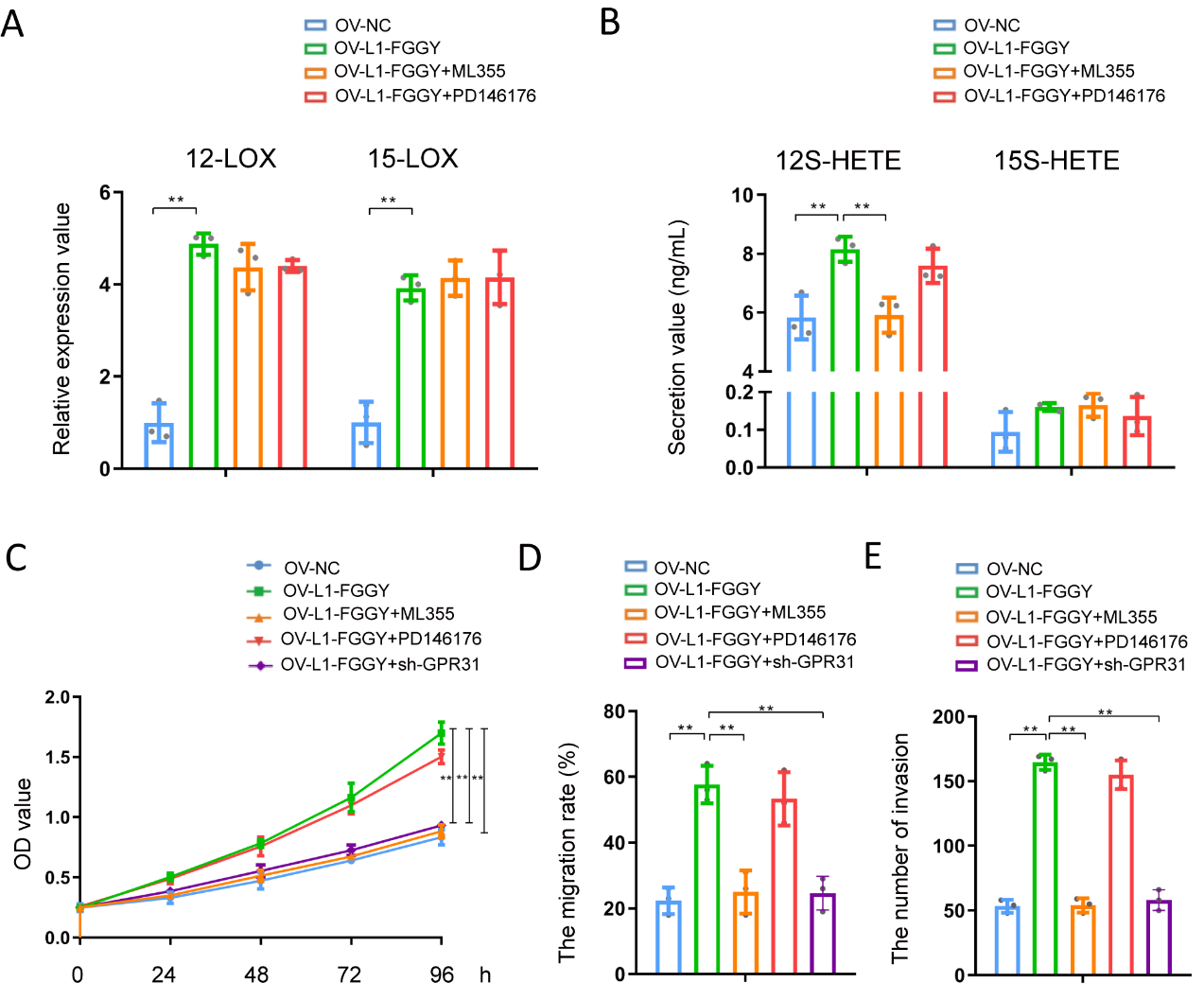


**Figure S7. The oncogenic roles of *L1-FGGY*/12-LOX/GPR31 in SK-MES-1 cells.** (A) The relative RNA expression of 12-LOX and 15-LOX detected in SK-MES-1^OV-CTRL^ and SK-MES-1^OV-^*^L1-FGGY^*, as well as in SK-MES-1^OV-^*^L1-FGGY^* treated with either ML355 or PD146176. (B) The secretion value of 12S-HETE and 15S-HETE detected in SK-MES-1^OV-CTRL^, SK-MES-1^OV-^*^L1-FGGY^*, and SK-MES-1^OV-^*^L1-FGGY^* treated with either ML355 or PD146176. (C) The proliferation of SK-MES-1^OV-CTRL^, SK-MES-1^OV-^*^L1-FGGY^*, SK-MES-1^OV-^*^L1-FGGY^*^+sh-^*^GPR31^*, and SK-MES-1^OV-^*^L1-FGGY^* treated with either ML355 or PD146176 was detected using CCK8 method. (D) The statistical results of migration rates of SK-MES-1^OV-CTRL^, SK-MES-1^OV-^*^L1-FGGY^*, SK-MES-1^OV-^*^L1-FGGY^*^+sh-^*^GPR31^*, and SK-MES-1^OV-^*^L1-FGGY^* treated with either ML355 or PD146176 in wound healing assays. (E) The statistical results of invasion number of SK-MES-1^OV-CTRL^, SK-MES-1^OV-^*^L1-FGGY^*, SK-MES-1^OV-^*^L1-FGGY^*^+sh-^*^GPR31^*, and SK-MES-1^OV-^*^L1-FGGY^* treated with either ML355 or PD146176 in trans-well invasion assays. The data are shown as mean ± SD with plots. * and ** indicate p<0.05 and p<0.01, respectively between the groups as indicated.


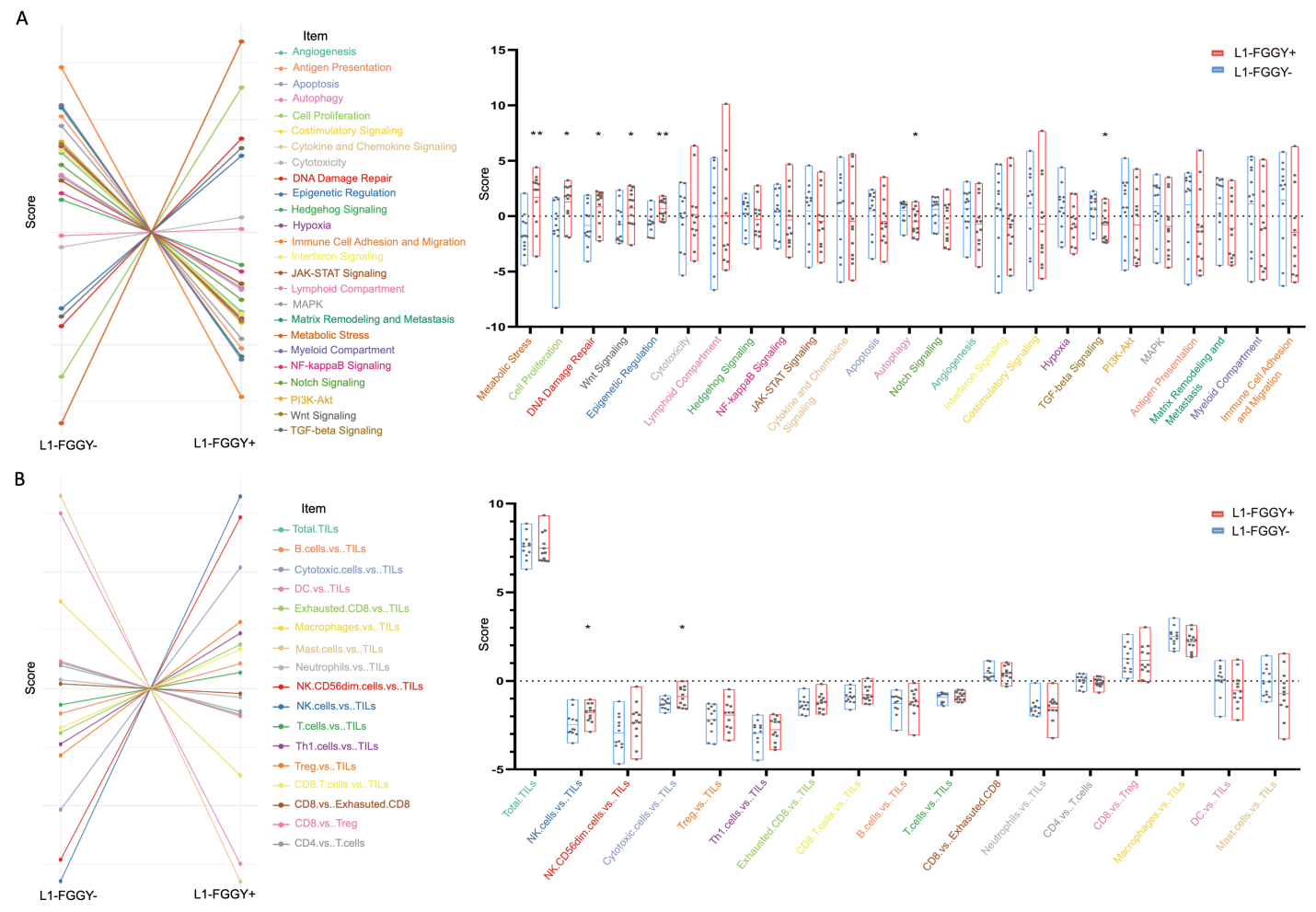


**Figure S8. The results of nCounter® PanCancer IO-360™ Panel detected in *L1-FGGY*^+^ tissues and *L1-FGGY*^-^ tissues (n=12).** (A) The pathway scores of different signaling pathways were compared between *L1-FGGY*^+^ tissues and *L1-FGGY*^-^ tissues. (B) The cell type scores of immunocytes were compared between *L1-FGGY*^+^ tissues and *L1-FGGY*^-^ tissues.


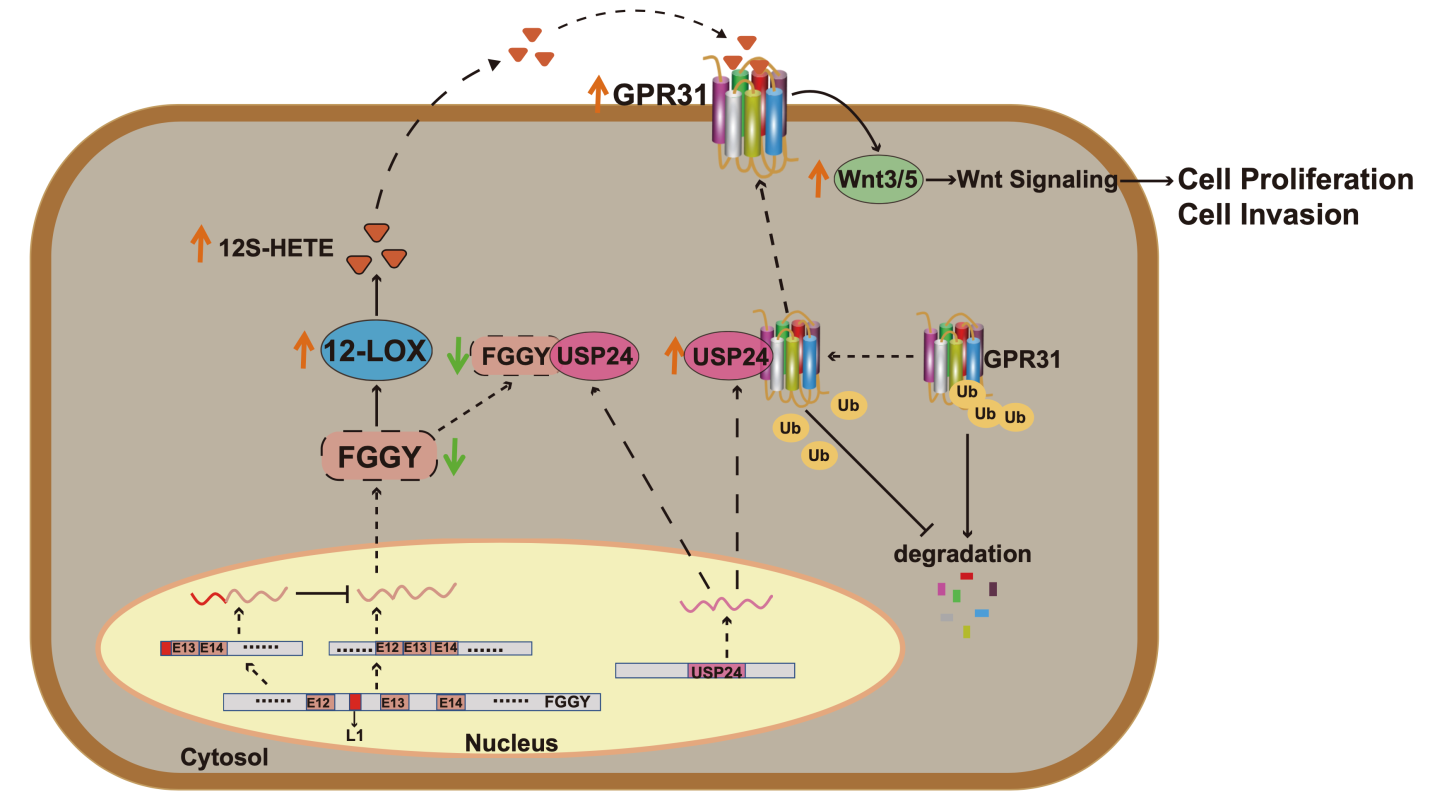


**Figure S9. The model of *L1-FGGY* activated 12-LOX/GPR31/Wnt signaling through competitive combination-induced USP24-mediated deubiquitination.**


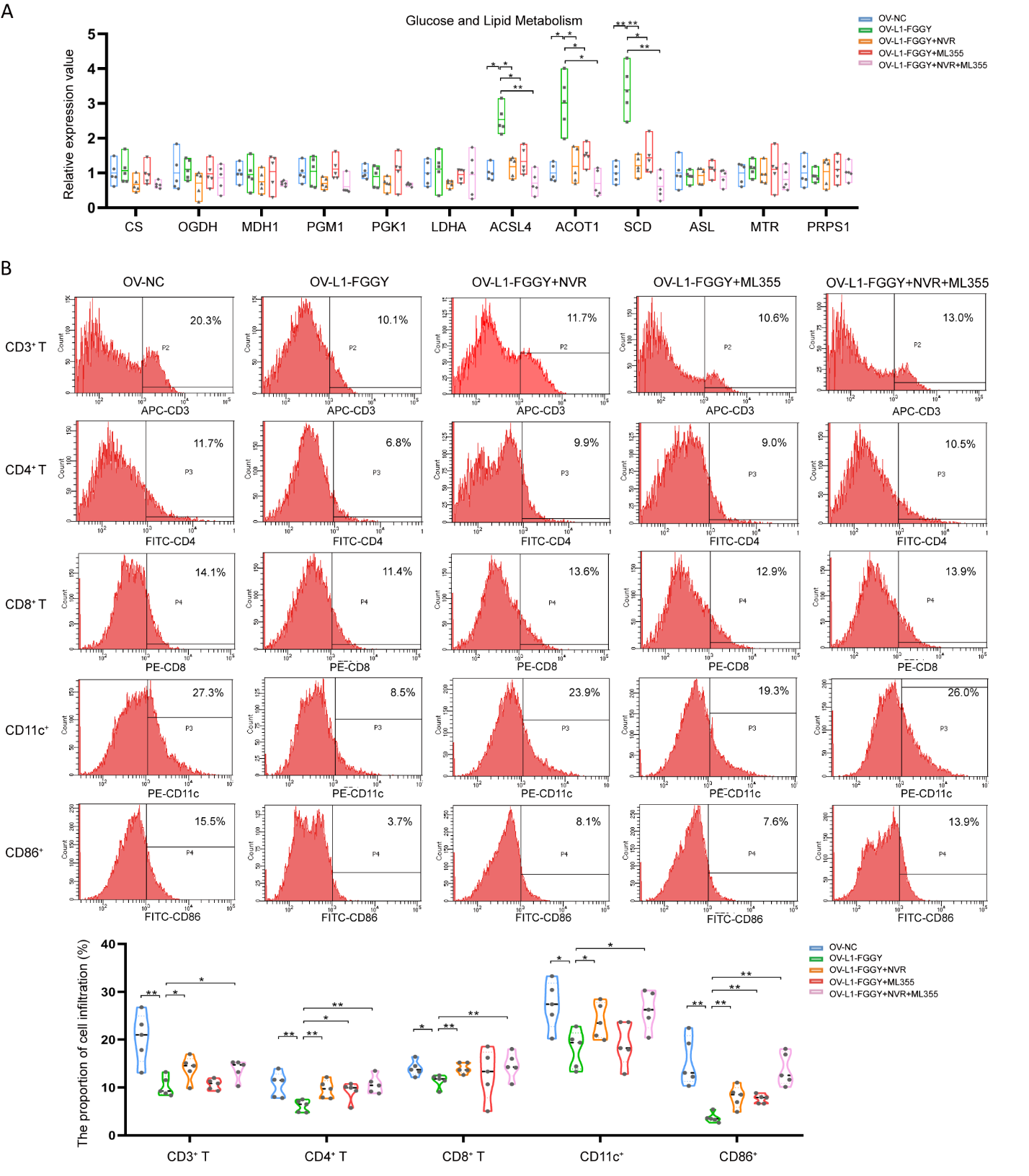


**Figure S10. NVR and ML355 triggered metabolism reprogramming and altered the immune microenvironment *in vivo*.** (A) The qPCR results of genes involved in glucose and lipid metabolism were shown as relative expression values in KLN205^OV-CTRL^ mouse group, KLN205^OV-^*^L1-FGGY^* mouse group, NVR-treated KLN205^OV-^*^L1-FGGY^* mice, ML355-treated KLN205^OV-^*^L1-FGGY^* mice and NVR+ML355-treated KLN205^OV-^*^L1-FGGY^* mouse group. (B) The proportions of various immunocytes in different mouse groups using flow cytometry.


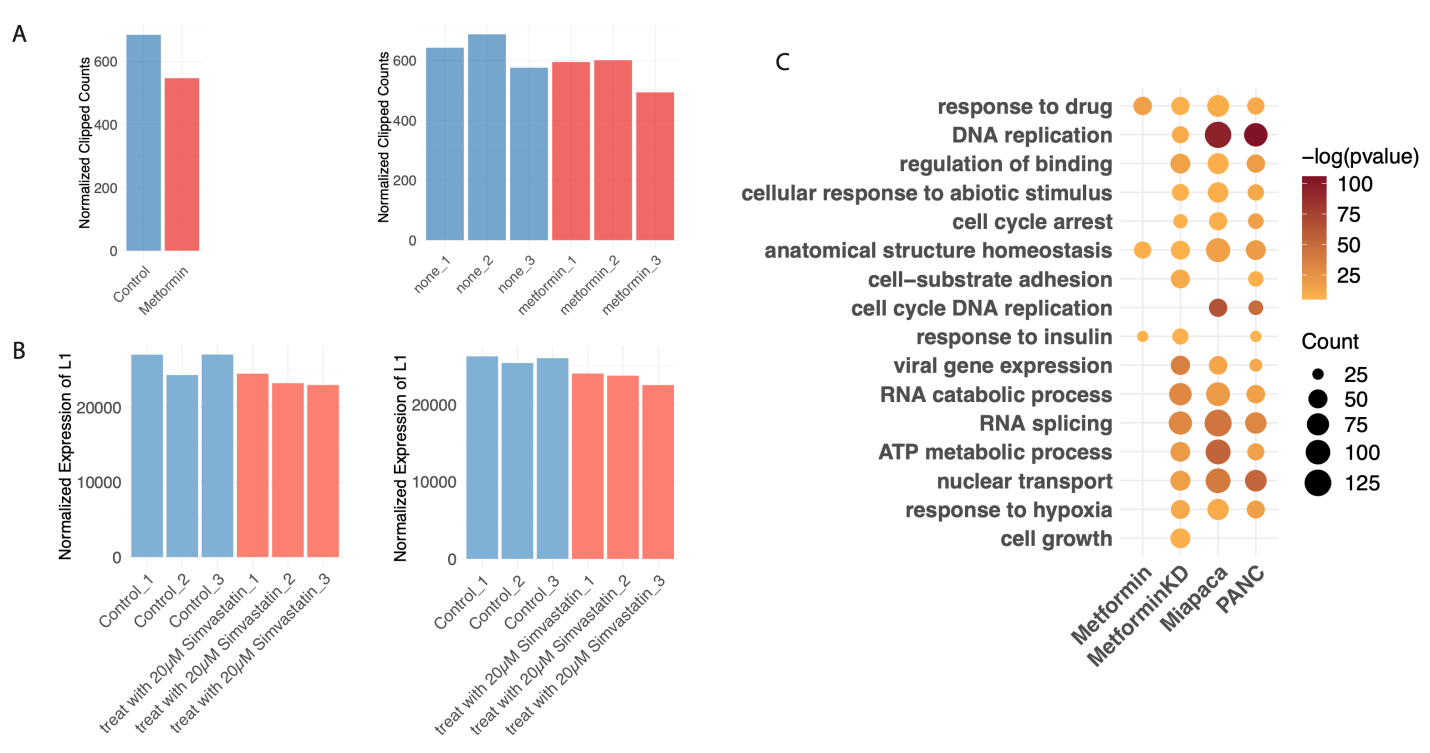


**Figure S11. Potential effects of drug on LCT activity.** (A) Distribution of LCT expression in samples treated with Metaformin and control samples in two public data sets. (GSE141052 and GSE146982) (B) Distribution of L1 expression in samples treated with Simvastatin and control samples in two pancreatic cancer cell line (PANC-1 and MiaPaCa-2 (GSE149566)).

**Table S1 Basic statistics of LCT events in all 3 cohorts.**

|  | LUSC | | LUAD | | Tianjin |  |
| --- | --- | --- | --- | --- | --- | --- |
|  | Case | Control | Case | Control | Case | Control |
| Number of Clipped Reads | 191.2 ± 159.48 | 68.19 ± 61.63 | 72.2 ± 63.89 | 23.54 ± 18.19 | 642.31 ± 295.16 | 204.14 ± 84.66 |
| Number of LCT Transcripts | 39.3 ± 26.55 | 20.31 ± 14.93 | 16.73 ± 9.7 | 9.7 ± 4.63 | 434.62 ± 162.46 | 158.36 ± 54.27 |
| Number of LCT affected Genes | 26.51 ± 16.29 | 16.54 ± 11.18 | 14.11 ± 7.85 | 8.44 ± 3.81 | 177.32 ± 49.44 | 97.59 ± 29.72 |

**Table S2 Overlapped LCT genes between TCGA LUSC and LUAD.**

**Table S3 Overlapped LCT genes between TCGA and TJMUCH LUSC samples**

**Table S4 Overlapped GO BP terms enriched with recurrent LCT genes between TCGA LUSC and LUAD.**

**Table S5 Enriched GO BP terms with recurrent LCT genes in TJMUCH cohort.**

**Table S6 GO BP terms enriched with genes positively co-expressed with overall LCT level in TCGA LUSC and LUAD samples.**

**Table S7 GO BP terms enriched with genes negatively co-expressed with overall LCT level in TCGA LUSC and LUAD samples.**

**Table S8 GO BP terms enriched with genes positively co-expressed with overall LCT level in TJMUCH LUSC samples.**

**Table S9 GO BP terms enriched with genes negatively co-expressed with overall LCT level in TJMUCH LUSC samples.**

**Table S10 Differentially expressed LCTs between tumor and normal samples in TCGA LUSC.**

**Table S11 Differentially expressed LCTs between tumor and normal samples in TJMUCH LUSC.**

**Table S12 Differentially expressed LCTs between tumor and normal samples in TCGA LUAD.**

**Table S13 Survival related LCTs in TCGA LUSC.**

**Table S14 Survival related LCTs in TCGA LUAD.**

**Table S15 Recurrent LCTs between bulk RNAseq from TCGA and 19 SC RNAseq samples.**

**Table S16 Differentially expressed genes between LCT enriched tumor cell cluster and other tumor cell clusters in each patient.**

**Table S17 GO BP enrichment of differentially expressed genes between LCT enriched tumor cell cluster and other tumor cell clusters in each patient.**

**Table S18 Differentially expressed genes between tumor cells with LCT versus tumor cells without LCT in LCT enriched tumor cell cluster in each patient.**

**Table S19 GO BP enrichment of differentially expressed genes between tumor cells with LCT versus tumor cells without LCT in LCT enriched tumor cell cluster in each patient.**

**Table S20. The basic clinical pathological information of all TJMUCH patients.**

| Clinical pathological parameters | Group 1 | Group 2 |
| --- | --- | --- |
|  | Number of patients | Number of patients |
| **Total** | 50 | 147 |
| **Gender** |  |  |
| Male | 46 | 119 |
| Female | 4 | 28 |
| **Age** |  |  |
| <60 years | 19 | 68 |
| ≥60 years | 31 | 79 |
| **Stage** |  |  |
| Ⅰ-Ⅱ | 38 | 94 |
| Ⅲ-Ⅳ | 12 | 53 |
| **T stage** |  |  |
| 1-3 | 45 | 136 |
| 4 | 5 | 11 |
| **N stage** |  |  |
| 0 | 33 | 90 |
| 1 | 8 | 21 |
| 2 | 9 | 36 |
| **M stage** |  |  |
| 0 | 50 | 135 |
| 1 | 0 | 12 |
| **Smoking** |  |  |
| Negative | 7 | 31 |
| Positive | 43 | 116 |

**Table S21. Distributions of clinical-pathological parameters of patients with *L1-FGGY*^+^ and *L1-FGGY*^-^ of TJMUCH**

| **linical pathological parameters** | Group 1 | | | Group 2 | | |
| --- | --- | --- | --- | --- | --- | --- |
|  | *L1-FGGY* | | *P* | *L1-FGGY* | | *P* |
|  | 0 | 1 |  | 0 | 1 |  |
| **No. of patients** | 22 | 28 | - | 77 | 70 | - |
| **Gender** |  |  | 0.621 |  |  | 0.011 |
| Male | 21 | 25 |  | 56 | 63 |  |
| Female | 1 | 3 |  | 21 | 7 |  |
| **Age** |  |  | 0.389 |  |  | 0.870 |
| <60 | 10 | 9 |  | 35 | 33 |  |
| ≥60 | 12 | 19 |  | 42 | 37 |  |
| **Stage** |  |  | 0.045 |  |  | 0.025 |
| Ⅰ-Ⅱ | 20 | 18 |  | 56 | 38 |  |
| Ⅲ-Ⅳ | 2 | 10 |  | 21 | 32 |  |
| **T stage** |  |  | 0.059 |  |  | 0.026 |
| 1-3 | 22 | 23 |  | 75 | 61 |  |
| 4 | 0 | 5 |  | 2 | 9 |  |
| **N stage** |  |  | 0.773 |  |  | 0.236 |
| 0 | 14 | 19 |  | 51 | 39 |  |
| 1-2 | 8 | 9 |  | 26 | 31 |  |
| **M stage** |  |  | 1.000 |  |  | 0.768 |
| 0 | 22 | 28 |  | 70 | 65 |  |
| 1 | 0 | 0 |  | 7 | 5 |  |
| **Smoking** |  |  | 0.035 |  |  | 0.008 |
| Negative | 6 | 1 |  | 23 | 8 |  |
| Positive | 16 | 27 |  | 54 | 62 |  |

**Table S22 List of 47 LCT related genes selected for grouping of patients.**

**Table S23. The sequences of primers in RT-qPCR analysis.**

| No. | Gene Name | Forward Primer | Reverse Primer |
| --- | --- | --- | --- |
| 1 | L1-FGGY | 5’-CGCCTTGCAGTTTGATCTCA-3’ | 5’-TGTGGCCAGGTAGAGAATGG-3’ |
| 2 | 5-LOX (human) | 5’-CCCGGGGCATGGAGAGCA-3’ | 5’-GCGGTCGGGCAGCGTGTC-3’ |
| 3 | 12-LOX (human) | 5’-CCATCTCACTGACCATTGTGG-3’ | 5’-CAGGCGGATGATGATGAGC-3’ |
| 4 | 15-LOX (human) | 5’-TTGGTTCTACTGGGTTCCTAATG-3’ | 5’-GGAGCCAAACGACATTTATCTG-3’ |
| 5 | Wnt3a (human) | 5’-AACTGCACCACCGTCCAC-3’ | 5’-AAGGCCGACTCCCTGGTA-3’ |
| 6 | Wnt5a (human) | 5’-GGTATATCACATGTCTCATTC-3’ | 5’-GGACTTTCCTCCCTGCTGCCC-3’ |
| 7 | β-catenin (human) | 5’-TGGTGACAGGGAAGACATCA-3’ | 5’-CCATAGTGAAGGCGAACTGC-3’ |
| 8 | TCF4 (human) | 5’-TGGGGGTGATTTTTTTTGGCTTT-3’ | 5’-GGTTCACGACGCTAAAGCTATTCT-3’ |
| 9 | CYP1A (mouse) | 5’-TGGTCGTGTCAGTAGCCAAT-3’ | 5’-TGAGCAGCTCTTGGTCATCA-3’ |
| 10 | CYP2B (mouse) | 5’-TCTGGCCACCATGAAAGAGT-3’ | 5’-GGGCCTCCTCCTTTATCCTC-3’ |
| 11 | 5-LOX (mouse) | 5’-GTTGGCATCTAGGTGCAGTG-3’ | 5’-CGGAATCGGATCATGGCTTC-3’ |
| 12 | 12-LOX (mouse) | 5’-CGGCCATGTTCAGTTGCTTA-3’ | 5’-GCACTTGGGATTCTCAGCAG-3’ |
| 13 | 15-LOX (mouse) | 5’-TACAAGACCGACCAAGCTGT-3’ | 5’-GATGAAGTGGCAAGCCTGAG-3’ |
| 14 | PTGS1 (mouse) | 5’-AATGCCACCTTCATCCGAGA-3’ | 5’-CTTTGGGTACAGAGGGCAGA-3’ |
| 15 | PTGS2 (mouse) | 5’-AAGCCTTCTCCAACCTCTCC-3’ | 5’-GCTGGGCAAAGAATGCAAAC-3’ |
| 16 | Wnt3a (mouse) | 5’-GTATGACAGTGCCTCGGAGA-3’ | 5’-TAGGTTCGCAGAAGTTGGGT-3’ |
| 17 | Wnt5a (mouse) | 5’-GAGTTCGTGGACGCTAGAGA-3’ | 5’-TACGGCCTGCTTCATTGTTG-3’ |
| 18 | β-catenin (mouse) | 5’-TATGGACTGCCTGTTGTGGT-3’ | 5’-TCGTGGAATAGCACCCTGTT-3’ |
| 19 | TCF4 (mouse) | 5’-CGTCTTCAGTCTACGCTCCT-3’ | 5’-AGGAGCTAGGGAAAGTGCTG-3’ |
| 20 | CS (mouse) | 5’-TGGCTGACACAGCTACAGAA-3’ | 5’-GACAGGAATAGCGAGGGTCA-3’ |
| 21 | OGDH (mouse) | 5’-GACAGCCTCCCAGACTGTTA-3’ | 5’-TCCAACCAGGCACAGTACAT-3’ |
| 22 | MDH1 (mouse) | 5’-AAGGCATGGAGAGGAAGGAC-3’ | 5’-GCTGGGTTTCCCACAACAAT-3’ |
| 23 | PGM1 (mouse) | 5’-CCTGCTCCAGAAGCAATCAC-3’ | 5’-GTCAAACTGCTGCTTTCCCA-3’ |
| 24 | PGK1 (mouse) | 5’-AAGACTGGCCAAGCTACTGT-3’ | 5’-TTGCTGCTCTCAGTACCACA-3’ |
| 25 | LDHA (mouse) | 5’-GCTGCTGATCGTCTCCAATC-3’ | 5’-AGCCTCTCTCCCATCAGGTA-3’ |
| 26 | ACSL4 (mouse) | 5’-CTTTGGAAGTGGCCTCACTG-3’ | 5’-AAAGCAAGTCTGTGCTGCAA-3’ |
| 27 | ACOT1 (mouse) | 5’-ATGACCAAGGATGGCCTCAA-3’ | 5’-TTCCACGGGAATGAAGCTCT-3’ |
| 28 | SCD (mouse) | 5’-TTCGTTAGCACCTTCTTGCG-3’ | 5’-GTGGTGGTAGTTGTGGAAGC-3’ |
| 29 | ASL (mouse) | 5’-TCTTCCCAGGGTACACACAC-3’ | 5’-ACATTGATCCGCTTCTGCAC-3’ |
| 30 | MTR (mouse) | 5’-ATGTGGCAGGATCCAGGAAA-3’ | 5’-ATCTGGTCATTGCACTGGGA-3’ |
| 31 | PRPS1 (mouse) | 5’-CTCCCACCAGGACTTATCCC-3’ | 5’-TTCGCCAATTTCCACACAGG-3’ |
| 32 | E-cadherin (mouse) | 5’-GACGTCCATGTGTGTGACTG-3’ | 5’-GCTCTTTGACCACCGTTCTC-3’ |
| 33 | N-cadherin (mouse) | 5’-GAAGGATGTGCACGAAGGAC-3’ | 5’-AGGGAAGCTTCTCACAGCAT-3’ |
| 34 | Snail (mouse) | 5’-TGGAAAGGCCTTCTCTAGGC-3’ | 5’-CTCTTCACATCCGAGTGGGT-3’ |
| 35 | Slug (mouse) | 5’-ACTACAGCGAACTGGACACA-3’ | 5’-GCCACTGGGTAAAGGAGAGT-3’ |
| 36 | Zeb1 (mouse) | 5’-CCACTGTGGAGGACCAGAAT-3’ | 5’-GTGAGGCCTCTTACCTGTGT-3’ |
| 37 | Twist1 (mouse) | 5’-GACTCCAAGATGGCAAGCTG-3’ | 5’-CCATCCTCCAGACGGAGAAG-3’ |
| 38 | β-actin | 5’-CTGAGAGGGAAATCGTGCGT-3’ | 5’-CCACAGGATTCCATACCCAAGA-3’ |
